# The Jaw Adductor Muscle Complex in Teleostean Fishes: Evolution, Homologies and Revised Nomenclature (Osteichthyes: Actinopterygii)

**DOI:** 10.1371/journal.pone.0060846

**Published:** 2013-04-02

**Authors:** Aléssio Datovo, Richard P. Vari

**Affiliations:** 1 Laboratório de Ictiologia, Museu de Zoologia da Universidade de São Paulo, São Paulo, Brazil; 2 Laboratório de Ictiologia de Ribeirão Preto, Department of Biologia, Universidade de São Paulo, Faculdade de Filosofia, Ciências e Letras de Ribeirão Preto, Ribeirão Preto, Sao Paulo, Brazil; 3 Department of Vertebrate Zoology, National Museum of Natural History, Smithsonian Institution, Washington, District of Columbia, United States of America; 4 Division of Fishes, Department of Vertebrate Zoology, MRC-159, National Museum of Natural History, Smithsonian Institution, Washington, District of Columbia, United States of America; Friedrich-Schiller-University Jena, Germany

## Abstract

The infraclass Teleostei is a highly diversified group of bony fishes that encompasses 96% of all species of living fishes and almost half of extant vertebrates. Evolution of various morphological complexes in teleosts, particularly those involving soft anatomy, remains poorly understood. Notable among these problematic complexes is the *adductor mandibulae*, the muscle that provides the primary force for jaw adduction and mouth closure and whose architecture varies from a simple arrangement of two segments to an intricate complex of up to ten discrete subdivisions. The present study analyzed multiple morphological attributes of the *adductor mandibulae* in representatives of 53 of the 55 extant teleostean orders, as well as significant information from the literature in order to elucidate the homologies of the main subdivisions of this muscle. The traditional alphanumeric terminology applied to the four main divisions of the *adductor mandibulae* – A_1_, A_2_, A_3_, and Aω – patently fails to reflect homologous components of that muscle across the expanse of the Teleostei. Some features traditionally used as landmarks for identification of some divisions of the *adductor mandibulae* proved highly variable across the Teleostei; notably the insertion on the maxilla and the position of muscle components relative to the path of the *ramus mandibularis trigeminus* nerve. The evolutionary model of gain and loss of sections of the *adductor mandibulae* most commonly adopted under the alphanumeric system additionally proved ontogenetically incongruent and less parsimonious than a model of subdivision and coalescence of facial muscle sections. Results of the analysis demonstrate the impossibility of adapting the alphanumeric terminology so as to reflect homologous entities across the spectrum of teleosts. A new nomenclatural scheme is proposed in order to achieve congruence between homology and nomenclature of the *adductor mandibulae* components across the entire Teleostei.

## Introduction

The infraclass Teleostei [1] is a speciose group of ray-finned fishes (Actinopterygii) encompassing more than 31,000 valid extant species with this total progressively increased by the annual description of hundreds of new species from both fresh and marine waters [2]. As one of the largest monophyletic lineages within the Vertebrata, teleosts encompass almost half of the known species-level diversity within that subphylum and include 99.8% of all extant bony fishes [2]–[4]. Teleosts demonstrate a remarkable repertoire of morphological modifications in all body systems. These reflect their adaptations to life in nearly all aquatic habitats from ocean depths to high mountain streams and the multiple alternative behavioral and reproductive strategies within this assemblage. Myriad researchers have engaged in anatomical explorations of teleosts over the centuries and contributed to our knowledge of the vast array of morphological adaptations within the group. Notwithstanding these endeavors, the evolution of many morphological complexes remains poorly understood across the infraclass. This limitation is particularly pervasive in the case of soft anatomical systems, including the skeletal musculature.

The *adductor mandibulae* usually is by far the most striking cranial muscle of teleosts [5], both in terms of proportional size and more significantly given its critical role in mouth functioning [6], [7]; an action central to respiration and food acquisition across all lineages. The bulk of the *adductor mandibulae* is composed of a massive facial segment positioned lateral to the suspensorium and usually connected anteriorly via tendinous tissue to a smaller mandibular segment of the muscle attached to be the medial surface of the lower jaw [5], [8]. Structurally the overall *adductor mandibulae* ranges from a simple, undivided muscle mass to an intricate architecture encompassing up to ten discrete subdivisions [5]. The ready access of the *adductor mandibulae* given its position on the lateral surface of the head and its pronounced plasticity across the spectrum of teleostean taxa resulted in this muscle being the focus of multiple studies. These analyses range across comparative morphology [8]–[19], phylogenetic reconstruction [20]–[30], ontogeny [31]–[37] and functional anatomy [6], [7], [38]–[44].

Although Owen [45], [46] previously proposed alternative nomenclatures for the teleostean *adductor mandibulae* complex, the terminology advanced by Vetter in 1878 [19] was applied in almost all subsequent myological studies through to the present. Vetter's [19] original nomenclature employed an alphanumeric naming convention in which the letter A (indicative of the *adductor mandibulae*) was combined with Arabic numbers and Greek letters. In combination these yielded a unique identifier for each of the subunits of the *adductor mandibulae* which Vetter encountered in the four teleosts he examined – the cypriniforms *Barbus* and *Cyprinus*, the esocoid *Esox* and the perciform *Perca*. The entire mandibular segment of the *adductor mandibulae* positioned medial to the lower jaw in these fishes was termed the Aω, whereas the main subdivisions of the facial segment located lateral to the suspensorium were designated as the A_1_, A_2_ and A_3_ sections. Under this identification system, the A_1_ section was a superficial muscle division inserting onto the maxilla, the A_2_ an external division attaching to the dorsal portion of the lower jaw and the A_3_ a more medially positioned component of the muscle inserting onto the inner aspects of the lower jaw proximate to the posterior terminus of Meckel's cartilage. Additional subdivisions of these main facial components were designated by the incorporation of a Greek letter as a suffix of the primary indicator for a particular section of the *adductor mandibulae* (*e.g.*, A_1_α, A_1_β).

Myological surveys involving the *adductor mandibulae* post Vetter [19] largely retained the essence of the terminology proposed by that author; however, the underlying evolutionary hypotheses of homology of muscle sections inherent in his nomenclature have long been generally ignored, either explicitly or implicitly. As a prime example, Vetter [19] postulated that the lateral facial sections of the *adductor mandibulae* (the A_1_ and A_2_ sections of his terminology) in the four teleosts examined in his study were derived from the more medially positioned A_3_. Subsequent studies based on broader surveys across teleosts alternatively proposed that A_3_ was derivative of A_2_ and eventually also lost in some taxa [5], [47]. Further complicating homology suppositions was the fact that some non-superficial facial divisions of the muscle were also designated as A_1_ or a subdivision of that muscle [18], [48], [49] as a consequence of their insertions on the maxilla. This practice directly conflicts with Vetter's original scheme under which A_1_ was a superficial portion of the *adductor mandibulae* with an insertion on the maxilla. Use of point of insertion on the maxilla as the overarching basis for homology hypotheses thereby resulted in the untenable assumption that positionally dramatically different muscles sections within the the *adductor mandibulae* (*i.e.*, on the lateral versus medial surfaces of the muscle) were, nonetheless, homologous.

Other minor alterations of the original terminology proposed by Vetter [19] include the designation of subdivisions of the main components of the muscle via the addition of superscript notation (*e.g.*, A_3_′ and A_3_″ [18]) and the substitution of Latin for Greek letters (*e.g.*, Aw for Aω [5]); this last procedure possibly being derived from the typographical restrictions inherent in some older publications. Finally, other authors advocated for the use of the path of the *ramus mandibularis trigeminus* nerve as a landmark useful for the purposes of identifying the facial divisions of the muscle [13], [16], [48], [50]–[53].

In retrospect, the traditional alphanumeric terminology proposed by Vetter [19] and slightly modified versions by some later authors most often proved applicable for comparative studies limited to small subgroups within the Teleostei (*e.g.*, [11], [16], [21], [29], [30], [54]). Contrarily, this terminology is patently inadequate when it comes to reflecting homologies of the components of the *adductor mandibulae* across the expanse of the Teleostei or for that matter often between many closely related orders within that infraclass. Inadequacy of the Vetter terminology for broad homology statements at higher phylogenetic scales has been long recognized by various researchers (Winterbottom, pers. comm.). As a consequence, even the most detailed and comprehensive synonymy of the teleostean skeletal muscles ever produced, that by Winterbottom [5], intentionally avoided advancing synonyms for the subdivisions of the *adductor mandibulae.* That author instead retained the alphanumeric terminology for descriptive purposes rather than as indicative of homology.

The present study centers on elucidating the morphological diversification of the *adductor mandibulae* in the Teleostei and identifying the homologies of its main components across that infraclass. In order to address these questions, we undertook a comparative analysis of the *adductor mandibulae* and its associated soft and hard anatomical structures in representatives of 53 of the 55 currently recognized orders of the Teleostei (only two rare monogeneric teleostean orders – Icosteiformes and Pholidichthyiformes – could not be included in the analysis) [1]. An extensive analysis of the literature was performed in order to summarize substantial comparative data and to evaluate both previous nomenclatural schemes involving the *adductor mandibulae* and prior hypotheses of evolution of the muscle across the infraclass.

The evidence demonstrates that the present alphanumeric nomenclature fails to identify homologous components of the *adductor mandibulae* across the Teleostei due to multiple factors discussed below. An alternative nomenclature that reflects these homologies across the entire Teleostei is proposed to facilitate this discussion along with future myologically based analyses in the infraclass.

## Materials and Methods

The classification of the Teleostei proposed by Wiley and Johnson [1] is employed herein. Nomenclature for the skeletal components of the neurocranium and lower jaw follows Patterson [55] and Nelson [56], respectively. Terminology for the elements of the suspensorium (*i.e.*, hyopalatine arch plus opercular series) follows Grande and Bemis [57] with the term palatine applied to the ossification resulting from the fusion of the autopalatine and dermopalatine or when a distinction between these two components is uncertain [58]. Cranial nerve terminology follows Freihofer [59].

Specimens that served for the analysis of the musculature were double-stained for cartilage and bone prior to dissection following the procedure outlined by Datovo and Bockmann [20]. Examined material (Table S1) is deposited in the following institutions: American Museum of Natural History, USA (AMNH); Laboratório de Biologia e Genética de Peixes, Universidade Estadual Júlio de Mesquita Filho, Brazil (LBP); Laboratório de Ictiologia de Ribeirão Preto, Brazil (LIRP); Museu de Zoologia da Universidade de São Paulo, Brazil (MZUSP); and National Museum of Natural History, Smithsonian Institution, USA (USNM). Access to the studied material of these collections was duly authorized by their respective curators. Specimens were examined in their original institutions or loaned to MZUSP or USNM.

Anatomical drawings were based on photographs and direct stereomicroscopic observations of specimens in order to capture fine anatomical details. Drawings are bidimensional and were all produced with a Wacom Intuos4 pen tablet (Wacom Company, Ltd., Tokyo, Japan). Outlines were generated in Adobe Illustrator CS5 and the shading and coloring in Adobe Photoshop CS5 (Adobe Systems, San Jose, CA, USA).

## Results

An enumeration of the invariant features that characterize the *adductor mandibulae* and related structures across a morphologically dramatically diverse group such as the Teleostei is difficult. The general features presented herein are intended to serve as guidelines to facilitate the recognition of the primary components of the muscle and associated soft tissues occurring in most teleosts and apparently reflect the myological patterns generalized for most teleostean orders. It is crucial to appreciate that these basic configurations are often altered among highly derived teleosts characterized by greatly restructured jaws with associated significantly modified musculature.

Universal descriptive guidelines for components of the *adductor mandibulae* that apply to all species of the morphologically and taxonomically diverse infraclass Teleostei are an unachievable goal. As is the case for virtually all morphological traits, an elucidation of the homologies of the components of the highly modified *adductor mandibulae* muscle can in many lineages be only achieved via comparisons with less derived but comparatively closely related taxa (*e.g.*, [8], [20], [22], [23]). Two additional systems, the buccal membranes and the *ramus mandibularis trigeminus* nerve, are intimately associated with the *adductor mandibulae* and pertinent to homology considerations. These are described in detail as appropriate.

### The buccal membranous system

Most teleosts have the *adductor mandibulae* associated directly or indirectly with the buccal membrane. In addition to lining the entirety of the oropharyngeal cavity, this membranous connective tissue complex interconnects the upper and lower jaws and suspensorium. Two primary components, in sum, form the buccal membrane: (1) a rostrolateral component termed the buccopalatal membrane [20] that is usually associated with the facial segment of the *adductor mandibulae*; and (2) a medially positioned posteroventral component termed the buccopharyngeal membrane occasionally associated with the intramandibular segment of the *adductor mandibulae*.

#### Buccopalatal membrane

**Figure 1:**
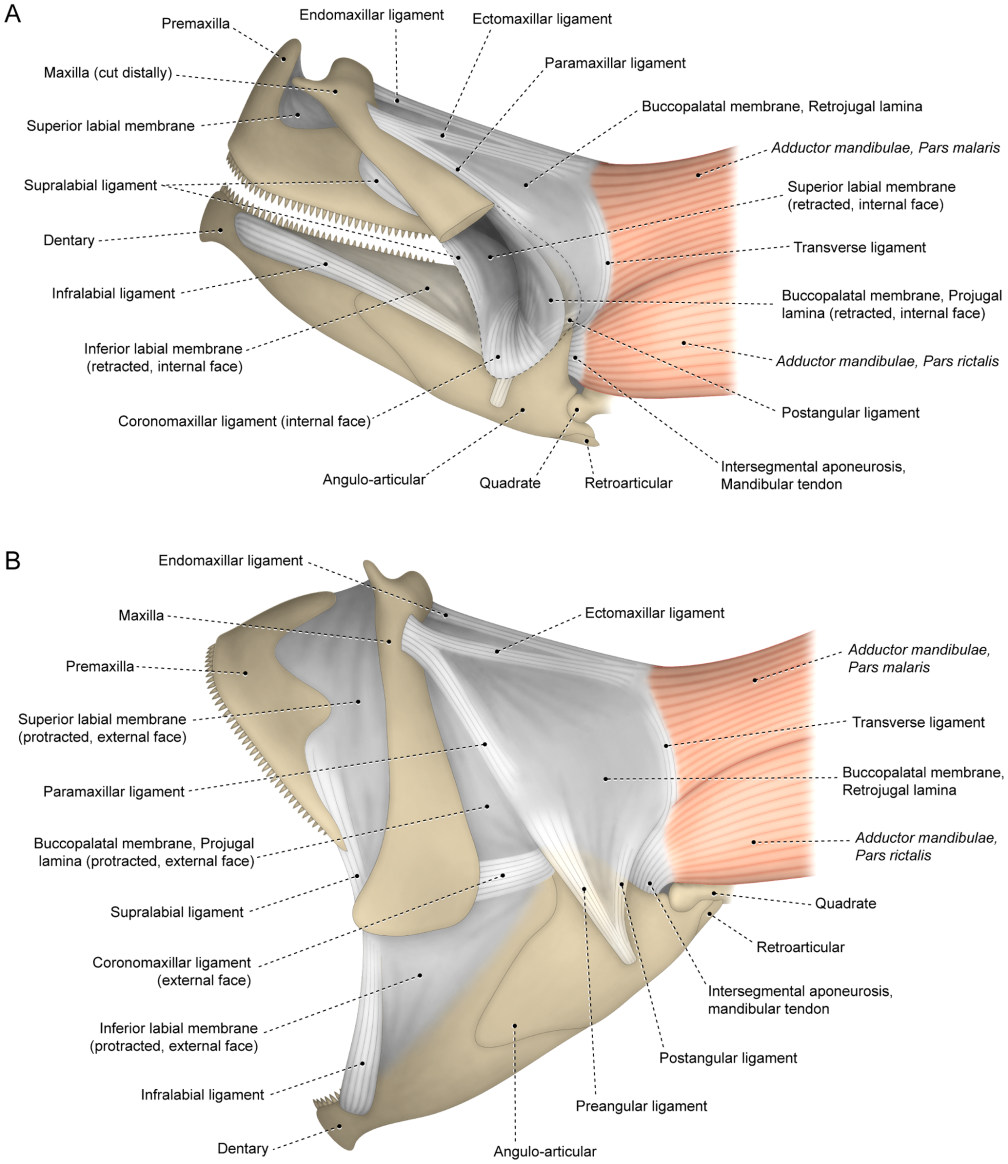
Buccopalatal membrane. Hypothetical teleost with protractile jaws exhibiting all nine main buccopalatal ligaments found across Teleostei. Left lateral view with mouth (A) closed and (B) open. Distal portion of maxilla cut in A, with dashed line representing outline of that bone.

The first of these major components of the buccal membrane, the buccopalatal membrane, forms the anterodorsolateral boundary of the buccal cavity. Ventrally, the buccopalatal membrane is limited by the lower jaw, anteriorly and anterodorsally by the premaxilla and maxilla and posteromedially by the anterodorsal margin of the suspensorium (Fig. 1). The buccopalatal membrane was most commonly quite obvious among examined teleosts but was on occasion present as an extremely thin membrane sometimes poorly differentiated from adjoining connective tissue systems. In some instances this results in the limits of this membrane being obscure in dissected specimens. Among a few teleostean groups the buccopalatal membrane is relatively simple and lacks obvious subdivisions [20]. Most teleosts conversely have a three-dimensionally complex buccopalatal membrane whose morphology significantly shifts during major mouth movements. In this more complex configuration the buccopalatal membrane usually has four main identifiable laminas or folds: the superior labial, inferior labial, projugal, and retrojugal laminas. It should be noted that these laminas together with the buccopalatal and buccopharyngeal membranes are almost invariably continuous. These subunits are chiefly intended herein as topographic descriptors to facilitate the following discussion.

The superior labial lamina extends between the posterior and posterodorsal margins of the premaxilla and the anterior and anteroventral margins of the maxilla (Fig. 1). As would be expected given the mobility and flexibility requisite for upper jaw motion and protrusion, the superior labial lamina is both most developed and demonstrates the greatest degree of expandability among those teleosts with protrusible premaxillae. The second of the laminae, the inferior labial lamina connects the anterodorsal border of the lower jaw to the distal portion of the maxilla and in some instances additionally to the premaxilla. In the course of mouth opening this lamina undergoes drastic changes in form as it progressively unfolds from its attachment area along the lower jaw to a largely flattened, completely unfolded configuration (Fig. 1B).

The portion of the buccopalatal membrane situated immediately posterodorsal to the maxilla similarly undergoes significant retraction and expansion in the course of the operation of the mouth. This portion, termed the projugal lamina [from the Latin *jugum*, an adjectival form meaning structures connected or yoked or pertaining to the cheek], is nearly invariably delimited posteriorly by the paramaxillar and preangular ligaments and ventrally by the coronomaxillar ligament (see below). In the closed mouth, the projugal lamina folds on itself and lies mostly internal to the retrojugal lamina (Fig. 1A) which is situated behind the projugal lamina in the open mouth (Fig. 1B). The retrojugal lamina, in turn, is the largest component of the buccopalatal membrane and is located just posterior to the projugal lamina from which it is usually separated by the paramaxillar and preangular ligaments. Dorsomedially the retrojugal lamina is attached to the ventral border of the anterodorsal portion of the suspensorium (usually to the autopalatine, ectopterygoid and quadrate). For the purposes of our study, the retrojugal lamina is the most significant component of the buccopalatal membrane given the frequent association of the posterior portion of this lamina with the facial segment of the *adductor mandibulae* muscle. In a few taxa the boundary between the superior and inferior labial laminas, as well as that between the projugal and retrojugal laminas, is difficult to discern in the maximally open mouth. Under such circumstances, it may be useful to employ the alternative terms of labial ( =  superior labial + inferior labial) and jugal ( =  projugal + retrojugal) laminas. Gosline [13] applied the term “primordial membrane” to a portion of the buccopalatal membrane. It remains unclear whether Gosline's [13] primordial membrane corresponds solely to what is herein termed the retrojugal plus projugal laminas or to a combination of those two laminas plus the inferior labial lamina of this study.

Forces generated during the opening and closing of the mouth and the application of pressure during feeding increase stresses in certain regions within the buccal membranes. Such additionally stressed regions likely eventually evolved into strengthened well-defined bands of collagen in the form of variably differentiated ligaments within the body of the membrane (Fig. 1) [7], [8], [13], [38], [60]. Degrees of differentiation of these buccal ligaments vary greatly across the Teleostei [13], with a spectrum of variably developed bands ranging from barely distinguishable ligamentous condensations within the lamina to well differentiated ligaments [8]. Much of the previous nomenclature applied to these ligaments parallels the problems discussed above for the alphanumeric terminology used for divisions of the *adductor mandibulae* muscle. Preeminent among these problems are: (1) the application of multiple names to a homologous structure in different taxa; (2) the use of the same name to designate non-homologous structures (*e.g.*, primordium or maxillo-mandibular ligament; see below); and (3) the failure to correctly identify the compound nature of structures resulting from the fusion of primitively separated ligaments. In order to resolve these problems and given the uncertainty inherent with the application of often poorly defined names, we avoid the use of ambiguous identifiers and introduce new standardized terminology for the buccal ligaments.

In several teleosts, portions of the *adductor mandibulae* associate with the buccal ligaments, which are thereby coopted to act as tendons of this muscle. Under the traditional definitions, a ligament interconnects two or more osseous structures, whereas a tendon joins a muscle to a bone, another muscle, or any other anchoring structure. The application of these standard definitions to the buccal ligaments would lead to the recognition of homologous structures via alternative qualifiers (ligament *vs.* tendon) in different taxa depending on the presence versus absence of a muscular association. As discussed by Johnson and Patterson [61], this inconsistency interjects ambiguity into comparative anatomical studies. Thus the usual convention of ligament versus tendon was herein superseded, when appropriate, to reflect homology hypotheses.

Nine discrete primary ligaments within the buccopalatal membrane were identified among examined teleosts (Fig. 1). By way of a preamble we emphasize (1) that most of the examined fishes only have a subset of the total suite of ligaments and (2) that apparently additional buccopalatal ligaments are present in some specialized groups of teleosts (*cf.* [62], [63], [64]). Ventrally, the retrojugal lamina attaches to the lateral face of the lower jaw where it usually has a reinforced attachment area on the posterior portion of the angular bone (or any compound ossification including the angular, such as the angulo-articular). Two ligaments may arise from this area of attachment: the preangular and postangular ligaments. The preangular ligament extends dorsally from its attachment on the angular towards the coronoid region of the retrojugal lamina where it may spread out over the surface of that lamina or alternatively fuse with the paramaxillar ligament (see below). The postangular ligament is relatively rare among teleosts and proceeds posterodorsally towards the posterior portion of the retrojugal lamina.

Three ligaments may be associated with the dorsal portion of the maxilla which typically is situated proximate to the mesethmoid (Fig. 1). The paramaxillar ligament arises from the posterodorsal region of the maxilla and runs posteroventrally almost parallel to this bone in the closed mouth, but with an acute separation from the margin of the maxilla in the open mouth. Distally, the paramaxillar ligament may dissipate into the body of the retrojugal lamina. When a preangular ligament is also present, its distal portion is often continuous with the posterior regions of the paramaxillar ligament. Although these two ligaments may remain separate from one another in some taxa (Fig. 1), a partial or total fusion of the paramaxillar and preangular ligaments is very common across teleosts. The resultant compound ligament, the preangulo-paramaxillar, has been previously referred to as the articular-maxillary [38], [39], [65], mandibulo-maxillare posterius [10], [47], [60], maxillo-dentary [66], maxillo-mandibular [28], [48], [54], [67], outer articulomaxillary [68] and primordial, primordiale, or primordium ligaments [5], [8], [13], [69], [70]. The preangulo-paramaxillar ligament is often associated with some of the facial sections of the *adductor mandibulae* muscle.

**Figure 2:**
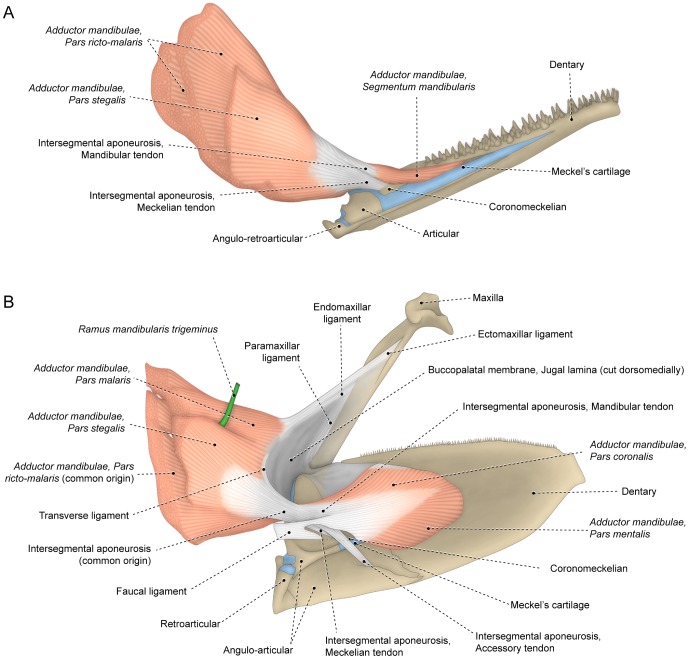
*Segmenta facialis* and *mandibularis* of the *adductor mandibulae*. Medial view of left muscle and associated structures of (A) *Hiodon tergisus* (Hiodontiformes: Hiodontidae; USNM 167970) and (B) *Poromitra capito* (Stephanoberyciformes: Melamphaidae; USNM 250603). Anteroventral region of faucal ligament cut to show accessory and meckelian tendons.

The ectomaxillar ligament arises from the anterolateral region of the maxilla (Fig. 1). When the paramaxillar and ectomaxillar ligaments co-occur in an individual, these bands may be continuous with one another anteriorly (see Yabe [29]: fig. 35C, E). From its area of attachment on the maxilla, the ectomaxillar ligament extends posteriorly to an area where it is usually associated with muscle fibers of the facial segment of the *adductor mandibulae* muscle. The endomaxillar ligament attaches to the medial surface of the dorsalmost portion of the maxilla. From that attachment area, this ligament proceeds posteriorly and becomes associated with the *adductor mandibulae* muscle (thus corresponding to the primordial ligament of Gosline [50]: fig. 2) and/or fuses with the posterior region of the ectomaxillar ligament.

The posteroventral margin of the retrojugal lamina is often continuous with the intersegmental aponeurosis; a tendinous complex that connects the mandibular and facial segments of the *adductor mandibulae* muscle (Fig. 1; see below). Several euteleosteomorphs possess a ligament arising from the intersegmental aponeurosis and running dorsally along the posterior border of the retrojugal lamina. This ligament, named the transverse ligament, usually serves as an attachment site for some fibers of the *adductor mandibulae*. In some taxa, the transverse ligament is partially or completely continuous with the posterior portions of the endomaxillar ligament and/or, rarely, also with the endomaxillar ligaments thereby forming a compound ligament.

The coronomaxillar ligament [20] is a short, stout ligament that attaches to the coronoid process of the lower jaw and the distal tip of the maxilla and marks the division between the retrojugal lamina and the inferior labial lamina (Fig. 1). The coronomaxillar ligament has been previously referred to as the anterior mandibulomaxillary [71], coronoid-maxilla [62], mandibulo-maxillare anterius [7], [60], mandibulo-maxillary [6], maxillomandibular [71], [72], maxillo-mandibulare anterius [47], maxillomandibulare mediale [70], maxillo-dentary [73], posterior maxillo-mandibular [64], or primordium [74] ligaments. The supramaxillary ligament of Howes [48] and what was termed a “non-osseous structure that resembles a supramaxilla” by Rosen and Patterson [75] also apparently correspond to a modified fibrocartilaginous coronomaxillar ligament (see below).

The infralabial ligament in the closed mouth is located along the anteroventral border of the inferior labial lamina, arises from the lateral surface of the anterior portion of the dentary and extends towards an attachment on the distal region of the maxilla (Fig. 1). This ligament may attach to, or merge into, the inferior labial lamina before directly attaching to the distal portion of the maxilla. The infralabial ligament of this study was termed the dento-maxillare by Osse [7].

The supralabial ligament extends from the posteroventral region of the premaxilla to the distal portion of the maxilla and forms the anteroventral border of the superior labial lamina. This ligament is often absent or poorly differentiated in examined taxa; a condition especially prevalent among basal teleosts. Terminology previously applied to this ligament includes the maxillary-premaxillary [48], maxillo-premaxillary [73], premaxilla-maxillary [76], premaxillomaxillare [70] and premaxillary-maxilla ligaments [77]. The posterior portions of the supralabial and infralabial ligaments may be conjoined (*e.g.*, in some atheriniforms, gadiforms and nandids), thus, forming a compound labial ligament that surrounds most of the gape of the mouth. The name labial ligament was previously applied by some authors to these combined ligaments [48], [78]–[82] but identifiers employed in other studies include the maxillomandibulare anterius [70] and maxillomandibulary [83].

The coronomaxillar, infralabial and on occasion the supralabial ligaments are sometimes very stout and fibrocartilaginous [7], [20], [60]. Fibrocartilage reinforcement of tendons and ligaments is considered an adaptation to offset compression [84], [85] or shear stress [86]. None of the coronomaxillar, infralabial and supralabial ligaments apparently undergo compression in the course of mouth motion but all of these ligaments, especially the coronomaxillar and infralabial, undergo wide rotational movements around their entheses (points of insertion onto their respective associated ossification) during jaw protrusion (Fig. 1). Such motions likely induce pronounced shear stresses.

Intensity of fibrocartilage reinforcement in the coronomaxillar and labial ligaments greatly varies across the Teleostei, ranging from the apparent total absence of fibrocartilage to such an extensive cartilaginous penetration of the ligament that the resultant structure is formed by superficial ligamentous sheets grading to a core consisting of true cartilage. This derived fibrocartilage reinforced form characterizes the coronomaxillar ligament of some siluriforms [20], the coronomaxillar and labial ligaments of some gadiforms [48], [75], and the labial ligaments of some atheriniforms [78], [80], [82], [87]. Based on their examination of a broad variety of fibrocartilage types, Benjamin and Ralphs [84] proposed that there exists “a continuous spectrum of tissues between dense fibrous connective tissue and hyaline cartilage”; a suggestion congruent with our findings on the different compositions of the coronomaxillar and labial ligaments across the Teleostei. In a further modified condition, the cartilaginous cores of these chondrified ligaments eventually ossify and on occasion may even support teeth. Cartilages of the supralabial and infralabial ligaments form the so-called maxillomandibulary and paradentary bones of dentatherinid and phallostethid atheriniforms, respectively [78], [80]–[83], [87]. A minute globular ossification apparently derived from the coronomaxillar was reported for the siluriform *Stauroglanis gouldingi* ( =  “unnamed submaxillary bone” of de Pinna [88]). These changes in the composition of the buccopalatal ligaments across the Teleostei constitute an interesting evolutionary sequence of morphological novelties in which connective membranes initially differentiate into ligaments which may subsequently chondrify, sometimes ossify and on occasion support dentition.

#### Buccopharyngeal membrane

The posteroventral portion of the buccal membrane is the buccopharyngeal membrane which is situated internal to the suspensorium and lower jaw. This membrane lines most of the buccopharyngeal cavity and connects the lower jaw and often the mandibular segment of the *adductor mandibulae* to the medial face of the suspensorium. A ligament may differentiate from the anteroventral portion of the buccopharyngeal membrane. When present, this ligament arises anteriorly from the mandibular segment of the *adductor mandibulae* and proceeds posteriorly to either progressively spread out over, and merge with, the buccopharyngeal membrane or more often to anchor to the medial face of the anteroventral bones of the suspensorium (the preopercle and/or more often the quadrate). This ligament is herein named the faucal ligament (from the Latin *fauces*, the posteriormost part of the buccal cavity leading into the pharynx).

### The adductor mandibulae muscle

The primary division of the *adductor mandibulae* in the Teleostei is into facial and mandibular muscle segments. These segments, termed the *segmentum facialis* and *segmentum mandibularis*, respectively, interconnect via a strong tendinous complex, the intersegmental aponeurosis [8]. In its simplest arrangement this aponeurosis is undivided but even in such a configuration a subtle differentiation can be perceived between the anterodorsal and anteroventral portions of the aponeurosis. The anterodorsal component – the mandibular tendon – serves as the site of origin for the *segmentum mandibularis* and the anteroventral component – the meckelian tendon – directly attaches anteriorly to the lower jaw (Fig. 2A). Contrarily, most other teleosts have the mandibular and meckelian tendons more obviously differentiated, with several additional subdivisions of the intersegmental aponeurosis distinguishable. Certain of these divisions are often continuous with subunits of the buccal membranes, thereby forming an intricate interoral ligamentous complex (Fig. 2B; [7], [40]). Degrees of differentiation of the tendons derived from the intersegmental aponeurosis vary, but these tendons are generally separated distally versus confluent and continuous with each other in the central portion of the intersegmental aponeurosis.

**Figure 3:**
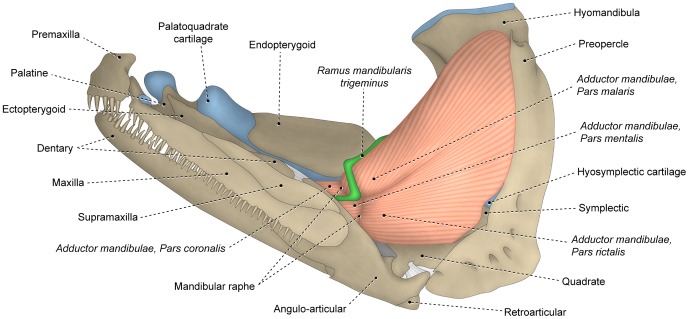
Differentiated facial sections with insertions solely on lower jaw. Lateral view of left *adductor mandibulae* muscle and associated structures of *Oncorhynchus mykiss* (Salmoniformes: Salmonidae; MZUSP 85378).

The mandibular tendon usually serves as the primary site of origin of the *segmentum mandibularis* of the *adductor mandibulae*. When present, the faucal tendon may be partially continuous anteriorly with the mandibular tendon (Fig. 2B). Several subgroups of the Teleostei (*e.g.*, some anabantiforms, argentiniforms, batrachoidiforms, beryciforms, cyprinodontiforms, nototheniiforms, percopsiforms, salmoniforms and stromateiforms) have the *segmentum mandibularis* expanded posteriorly and directly contacting the anterior portion of the *segmentum facialis*. In such cases a raphe marks the limits between the *segmenta mandibularis* and *facialis*. This raphe, herein termed the mandibular raphe, is always continuous medially with the mandibular tendon (Fig. 3).

The meckelian tendon (Fig. 2) is usually transversely flattened posteriorly, but becomes gradually more cylindrical as it proceeds anteriorly towards its attachment on the medial face of the lower jaw proximate to the posterior margin of Meckel's cartilage. The coronomeckelian bone forms ontogenetically as an ossification of the distal tip of the meckelian tendon [89]–[93] and serves as the site of attachment for the meckelian tendon (Fig. 2) in the vast majority of the examined taxa.

In various teleosts, the intersegmental aponeurosis gives rise anteriorly to a third tendinous branch herein termed the accessory tendon which usually passes medial to the meckelian tendon and posterior to the mandibular tendon. Distally, the accessory tendon may dissipate within the *segmentum mandibularis* (some anabantiforms) or attach to several of the components of the lower jaw including the medial portion of the coronomeckelian bone (some elopiforms and salmoniforms), the ventral region of the dentary (some characiforms [8] and stromateiforms), or more often the ventral portion of the angulo-articular (some characiforms [8] and most neoteleosts; Fig. 2B). Among many teleosts, the accessory tendon arises from the main body of the mandibular tendon (*e.g.*, some anabantiforms, characiforms, cypriniforms, stromateiforms and trachiniforms). Based on this configuration, Datovo and Castro [8] interpreted the accessory tendon of characiforms as a subdivision of the mandibular tendon and named it the mandibular accessory tendon. Examination of a greater diversity of teleosts reveals that this tendon alternatively may share a common origin solely with the meckelian tendon (*e.g.*, polymixiforms) or even arise independent of both the meckelian and mandibular tendons (*e.g.*, some gadiforms, perciforms and stephanoberyciforms; Fig. 2B). In light of this broad range of origins and in order to highlight its independence from the mandibular tendon, the structure previously named the mandibular accessory tendon [8] is herein renamed the accessory tendon. This tendon was identified in several not closely related teleostean groups and, consequently, it may have evolved and/or was lost independently in several lineages.

**Figure 4:**
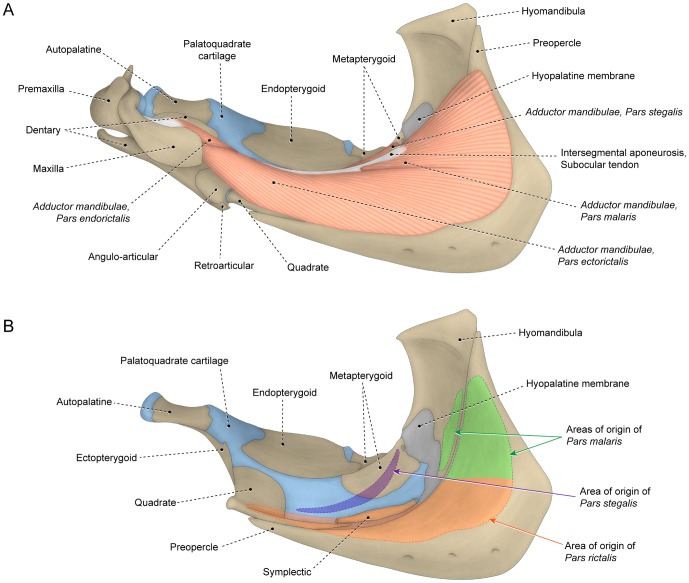
Differentiated facial sections with *rictalis* inserting on upper jaw. Lateral view of left (A) *adductor mandibulae* and associated structures and (B) sites of origin of *segmentum facialis* on suspensorium of *Chanos chanos* (Gonorynchiformes: Chanidae; USNM 173572).

Posteriorly the intersegmental aponeurosis may be expanded and subdivided in a mode comparable to the anterior portion of that connective tissue band, albeit with these subdivisions less common and less significant for the purposes of our discussion. A posterodorsal branch of the intersegmental aponeurosis, the subocular tendon, runs along the dorsal rim of the *segmentum facialis* and conforms to the contour of the posteroventral margin of the eyeball [8] (Fig. 4A). The subocular tendon restricts compression and deformation of the eyeball during contraction of the *adductor mandibulae* [94], [95]. Not surprisingly, this tendon is better developed in those teleosts with comparatively large eyes immediately juxtaposed to the adjacent *adductor mandibulae* [8], [95]. Other tendinous bands along the area of contact of the *segmentum facialis* with the eyeball, but not derived from the intersegmental aponeurosis (thus, not homologous to our subocular tendon), may also be present. The facial tendon is a posteroventral division of the intersegmental aponeurosis that parallels the ventral border of the *segmentum facialis* and attaches to the ventrolateral surface of the suspensorium, usually onto the quadrate. The facial tendon is known only in some aulopiforms, characiforms [8] and stomiatiforms.

It is worthy of note that the aforementioned tendons derived from the intersegmental aponeurosis may associate with different muscle sections in different teleostean groups. Some associations are conversely highly conserved in various cases as exemplified by the invariable association of the meckelian tendon with the *stegalis* (see next section).

#### Segmentum facialis

**Figure 5:**
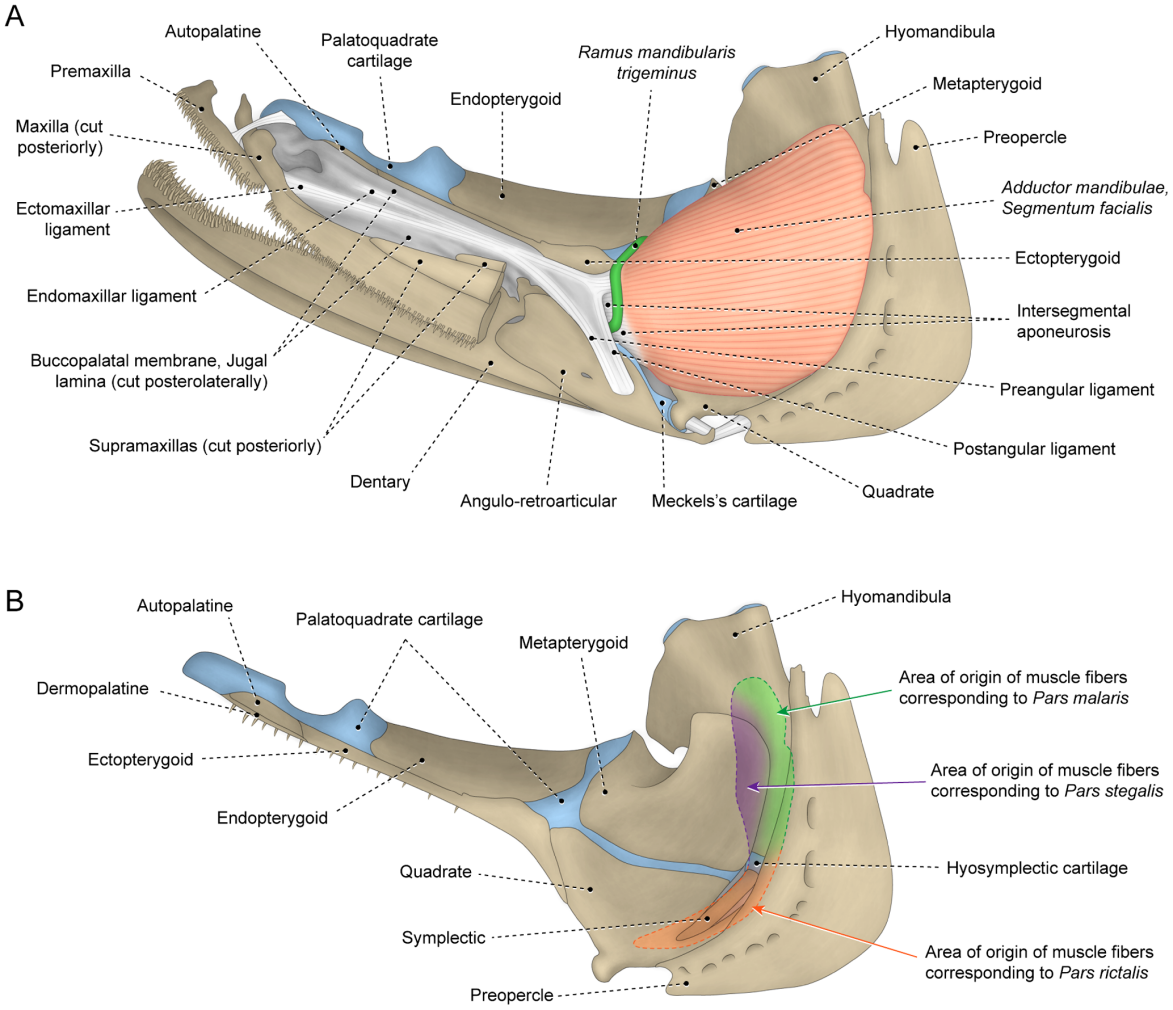
Undifferentiated facial sections with insertion on retrojugal lamina and lower jaw. Lateral view of left (A) *adductor mandibulae* and associated structures and (B) sites of origin of *segmentum facialis* on suspensorium of *Elops lacerta* (Elopiformes: Elopidae; MZUSP 84787).

**Figure 6:**
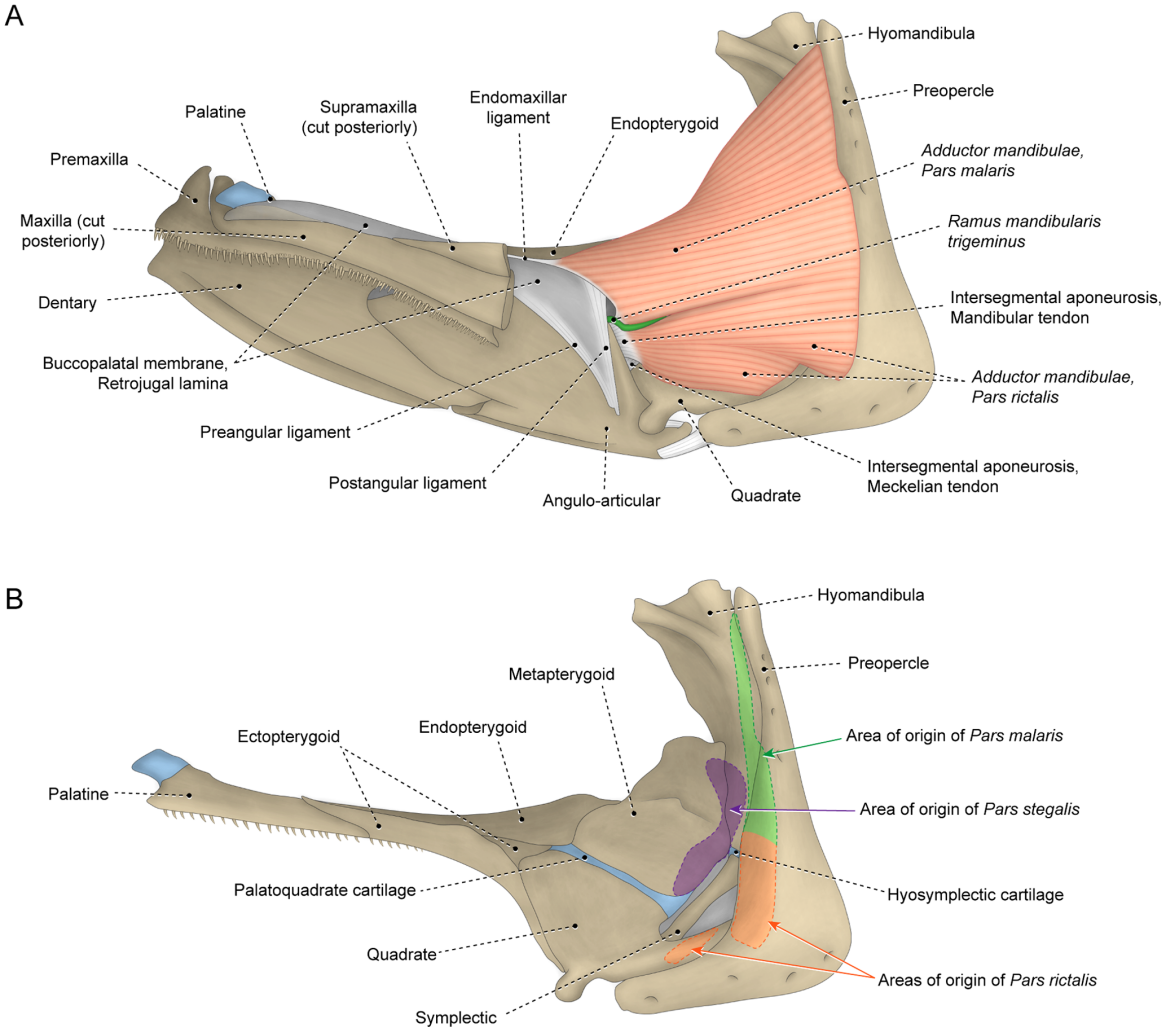
Differentiated facial sections with *malaris* inserting on upper jaw. Lateral view of left (A) *adductor mandibulae* and associated structures and (B) sites of origin of *segmentum facialis* on suspensorium of *Hime japonica* (Aulopiformes: Aulopidae; USNM 384078).

The *segmentum facialis* of the *adductor mandibulae* is situated on the cheek and originates primarily from the lateral surface of various elements of the suspensorium (usually the preopercle, hyomandibula, quadrate and metapterygoid; Fig. 5B), although the neurocranium and the medial face of the infraorbital series may also serve as sites of origin. In some basal teleosts such as the elopomorph *Elops* (Fig. 5B) and the otomorph *Denticeps*, the *segmentum facialis* lacks any trace of differentiation or subdivision. Alternatively, in most other teleosts, the *segmentum facialis* is differentiated and often subdivided into subunits regardless of whether it inserts solely on the lower jaw (Fig. 3) or onto both the upper and lower jaws (Figs. 4A, 6A). Three main subunits of the *segmentum facialis* are usually identifiable, albeit with the degree of separation of these portions highly variable and ranging from completely independent units to continuity across the totality of the sections. The three primary subunits of the *segmentum facialis* are herein referred to as sections or *partes* (singular *pars*) and are named *rictalis*, *malaris* and *stegalis*. Therefore, the terms section or *pars* of a muscle refers to any identifiable muscular subunit whose homology and evolutionary history can be traced and studied across the examined taxa regardless of the degree of separation/differentiation between that and other sections.

It is notable that many teleosts lack a definitive separation between the *rictalis*, *malaris* and *stegalis* sections although a differentiation between these sections is readily apparent. For example, the osteoglossomorph *Hiodon* has all the facial sections fully continuous with one another but the *stegalis* is unambiguously differentiable from the remaining sections of the *segmentum facialis* by its more anterior area of origin (Fig. 2). In the protacanthopterygian *Oncorhynchus*, the differentiation between the *malaris* and *rictalis* is most obviously evidenced by differing orientations of their superficial muscle fibers (Fig. 3). In other teleosts, the three primary facial sections are distinctly separated from each other (Figs. 4A, 6A). Separation and/or differentiation between the sections of the *segmentum facialis* (*rictalis*, *malaris* and *stegalis*) may be total (*i.e.*, along their entire extent) or partial (restricted to a portion of the muscle). Often, some facial sections are continuous with each other at their origin but gradually differentiated (Fig. 3) or separated (Figs. 4A, 6A) towards their insertions.

It is critical to appreciate that the muscle sections detailed below are subdivisions of the *segmentum facialis*. Recognition of this identity is crucial for understanding the course of evolution of this muscle across the Teleostei. Indeed, one can argue that a failure to appreciate the homology of the following muscles with specific parts of the *segmentum facialis* underlies much of the confusion involving the homologies and nomenclature of the sections of the *adductor mandibulae* (see Discussion).

#### Pars rictalis

The lateral portion of the *segmentum facialis* is composed of two primary sections; a ventral component termed the *pars rictalis* and a dorsal element named the *pars malaris* (Figs. 3, 4A, 6A). The *rictalis* section originates from the ventrolateral region of the suspensorium with fibers usually attaching onto the ventrolateral portions of the quadrate and the anteroventral portion ( =  the horizontal arm) of the preopercle (Figs. 4B, 6B). Sites of insertion of the *rictalis* section are variable. In most teleosts this section inserts on the lower jaw via an intersegmental aponeurosis and/or *segmentum mandibularis* (Figs. 3, 6A). The *rictalis* attaches directly to the lower jaw bones in a few examined groups (*e.g.,* some ostariophysans, blenniiforms, cottiforms, labriforms and protacanthopterygians). Many ostariophysans, smegmamorpharians, anabantiforms, gobiesociforms and a few perciforms have the *rictalis,* or a part of that section, inserting onto the maxilla (Fig. 4A). In most cases, the connection with the maxilla is achieved via the retrojugal lamina and/or its embedded ligaments. Notwithstanding this variation in the areas of the insertion of the muscle section, the apex of the *rictalis* is almost always located near to the corner of the mouth [ =  *rictus*, in Latin] with that position being the basis for its name.

In some of the examined taxa (*e.g.,* some acanthuriforms, anabantiforms, gobiiforms, ostariophysans and smegmamorpharians), the *rictalis* is differentiated into an external subsection, herein termed the *ectorictalis*, and an internal subsection, named the *endorictalis* (Fig. 4A). These subsections are often only partially separated from each other. In many of the taxa with a divided *rictalis*, one of the subsections inserts onto the maxilla and the other onto the lower jaw.

#### Pars malaris

The *pars malaris* forms the dorsolateral portion of the *segmentum facialis* and is located immediately posteroventral to the eyeball (Figs. 3, 4A, 6A). The *malaris* is usually the most massive component of the *adductor mandibulae* and occupies a large portion of the cheek [ =  *mala*, in Latin]. The *malaris* arises from the posterodorsal region of the suspensorium, usually on the lateral surfaces of the hyomandibula and the posterodorsal portion ( =  vertical arm) of the preopercle (Figs. 4B, 6B). As in the case of the *rictalis*, the insertion of the *malaris* is notably variable. In most of the lower teleosts (*i.e.,* non-neoteleosts), smegmamorpharians, anabantiforms and a few perciforms, the *malaris* inserts primarily or exclusively on the lower jaw via the intersegmental aponeurosis (Figs. 3, 6A). In several of these lower teleosts, the *malaris* (or the muscle portion corresponding to it – *i.e.*, the dorsolateral fibers of the *segmentum facialis*) also inserts on the posterodorsal region of the retrojugal lamina. This condition is found, for example, in the elopomorphs *Elops* (Fig. 5) and *Megalops*, the otomorph *Pellona* and the protacanthopterygian *Osmerus*. In most neoteleosts, the *malaris* is expanded anterodorsally and more intimately associated with the retrojugal lamina and the embedded ligaments leading to the maxilla, especially the endomaxillar ligament (Figs. 2B, 6A). In spite of the increased association of the *malaris* with the maxilla in neoteleosts, the ventral portion of the *malaris* in these fishes usually retains a connection with the intersegmental aponeurosis and, consequently, with the lower jaw (Figs. 2B, 6A). This connection is lost and the *malaris* becomes solely associated anteriorly with the maxilla in a relatively few taxa (some acanthuriforms, aulopiforms, batrachoidiforms, gadiforms and myctophiforms).

**Figure 7:**
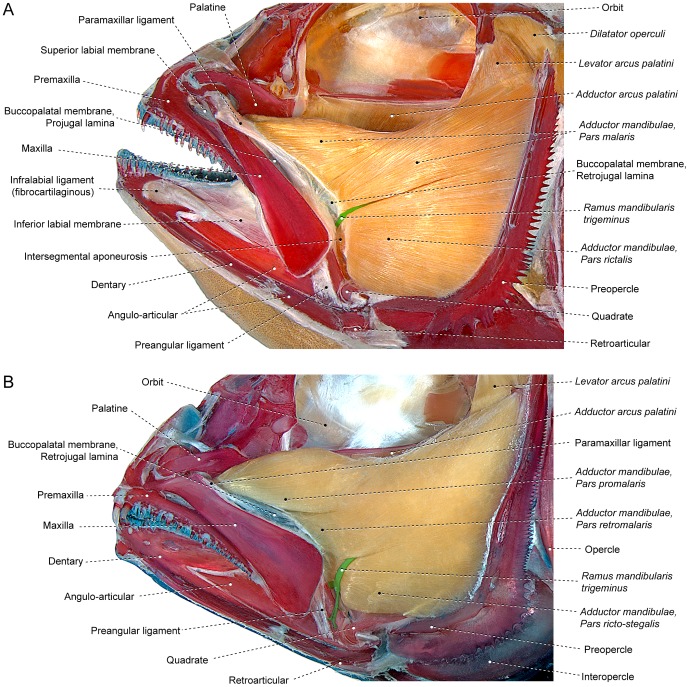
Expansion of *malaris* over retrojugal lamina. Lateral view of left *adductor mandibulae* and associated structures of (A) *Dules auriga* (Scorpaeniformes: Serranidae; MZUSP 70831) and (B) *Lutjanus analis* (Perciformes: Lutjanidae; LIRP 1866).

**Figure 8:**
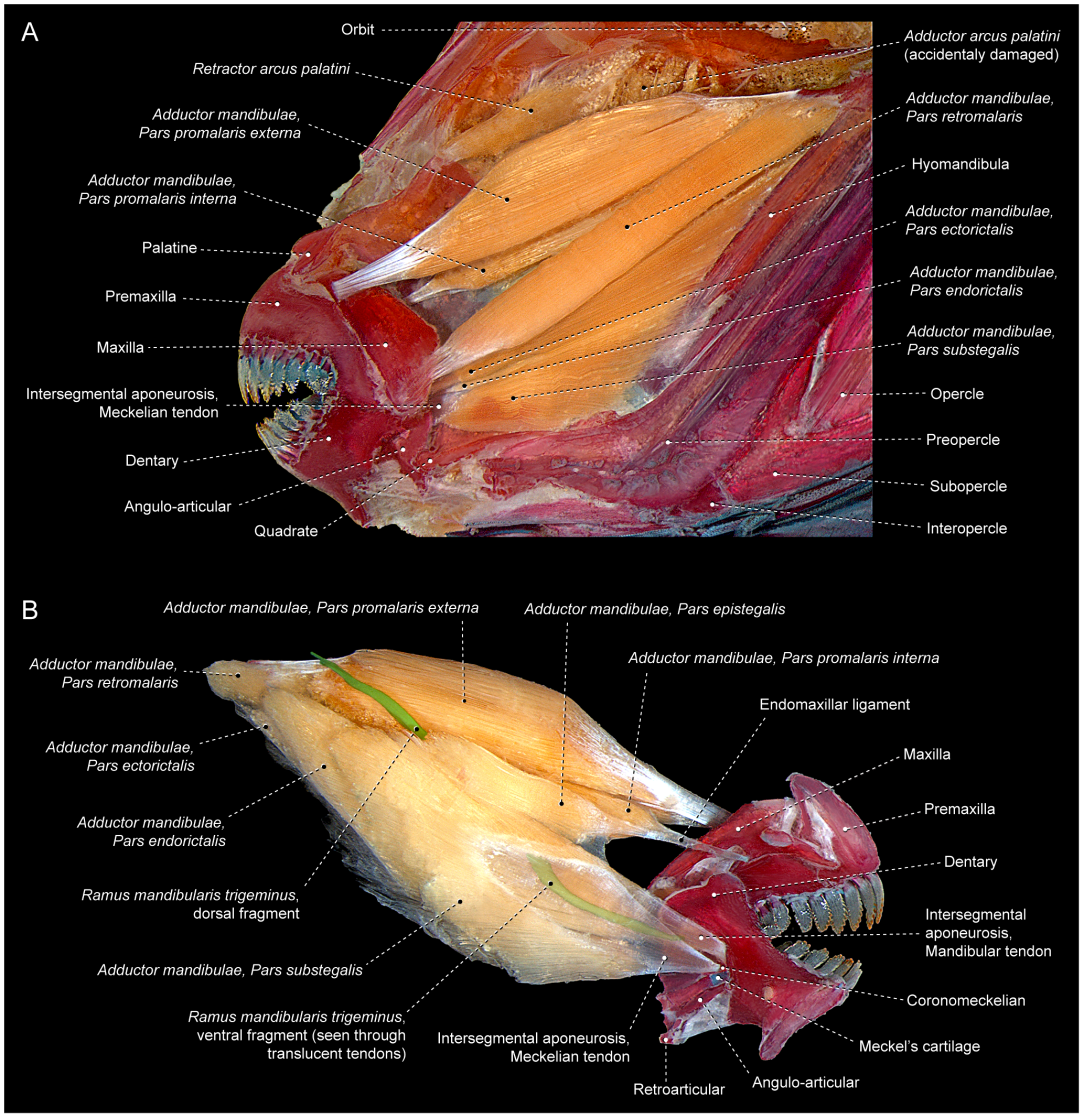
Highly subdivided *segmentum facialis*. Left *adductor mandibulae* and associated structures of *Acanthurus chirurgus* (Acanthuriformes: Acanthuridae; MZUSP 48207) in (A) lateral and (B) medial view. *Ramus mandibularis trigeminus* digitally colored in green.

The anterior expansion of the *malaris* over the retrojugal lamina is yet more pronounced in some neoteleosts in which the muscle nearly directly reaches the maxilla (*e.g.*, *Dules,* Serranidae; Fig. 7A). In several taxa, this process ultimately leads to the anterior differentiation of this section of the muscle into two subunits: a posteroventral *retromalaris* that usually inserts on the posterolateral region of the retrojugal lamina proximate to the conjunction of the preangular and paramaxillar ligaments; and an anterodorsal *promalaris* that tapers anteriorly towards the anterodorsal region of the retrojugal lamina and becomes primarily associated with the endomaxillar ligament and on occasion additionally with the ectomaxillar ligament (*e.g.*, *Lutjanus*, Lutjanidae; Fig. 7B). Differentiation between the *promalaris* and *retromalaris* sections is often restricted to the anterior portion of the muscle; a morphology present in some carangiforms, perciforms (Fig. 7B) and scombriforms. A complete separation of the *promalaris* from the *retromalaris* occurs in some acanthuriforms, gadiforms, percopsiforms, ophidiiforms, scorpaeniforms and tetraodontiforms in which the division between those sections extends posteriorly to their origins (Fig. 8). The plane of the primary division between the *promalaris* and *retromalaris* may progressively shift along the anteroposterior extent of the muscle; changing from running along a nearly horizontal alignment proximate to the insertions of these sections to a nearly vertical plane in the region of their origins. As a consequence, the origin of the *promalaris* may sometimes lie fully medial to the origin of the *retromalaris*.

#### Pars stegalis

The *stegalis* [from Greek *stego*, meaning hidden, covered] is the innermost component of the *segmentum facialis* and is mostly, or completely, covered laterally by the *malaris* and *rictalis* portions of that muscle (Fig. 2). The fibers of the *stegalis* often extend posteriorly for a shorter distance than do the fibers of the other facial sections resulting in a more anterior origin of that section. Sites of origin of the *stegalis* are the lateral surfaces of the metapterygoid and often the anterior portion of the hyomandibula (Figs. 4B, 6B). In the vast majority of the teleosts, the entirety or at least the ventral portion of the *stegalis* converges onto the meckelian tendon or the ventral portion of the intersegmental aponeurosis which, in turn, anteriorly differentiates into a meckelian tendon. As mentioned above, the meckelian tendon invariably inserts on the lower jaw, usually on the coronomeckelian bone. The distinctly anteriorly displaced origin of the *stegalis*, which almost always involves the metapterygoid, and/or the association of the section with the meckelian tendon, allows for the unequivocal recognition of the *stegalis* in all examined teleosts, even when it is largely continuous with another facial section of the *adductor mandibulae* (Fig. 2A).

Several examined teleosts have the dorsal portion of the *stegalis* somewhat differentiated from the ventral region of the section. In some cases, this differentiation is apparently a function of the fact that the dorsal portion of the *stegalis* originates medial to the *levator arcus palatini*. This differentiation becomes more pronounced in various taxa in which the *stegalis* becomes at least partially divided into an anterodorsal *epistegalis* and a posteroventral *substegalis*. Some acanthuriforms, siluriforms and tetraodontiforms demonstrate a further modified condition in which the *epistegalis* acquires an insertion on the maxilla, while the *substegalis* retains its association with the meckelian tendon and the lower jaw (Fig. 8). Among some siluriforms exhibiting this arrangement, the *epistegalis* has been termed the *retractor tentaculi*; a name alluding to its presumed function in maxillary barbel retraction among these fishes. The name *retractor tentaculi* has, however, been applied to various muscle sections in addition to the *epistegalis*; all of which share in common an attachment to the maxilla [37].

#### Compound facial sections

In several instances, two or more facial sections are continuous with each other to significant degrees thereby forming a compound muscle section. A compound *ricto-malaris* is more commonly found in non-neoteleosts (Fig. 2A) whereas a *ricto-stegalis* is more frequent in neoteleosts. The *stego-malaris* is known to occur, in turn, among some ostariophysans and smegmamorpharians. Other different combinations of sections and subsections such as the *endoricto-malaris* of some cypriniforms have also been observed. It is critical to note that two or more sections may be separated or differentiated at one extremity of the muscle (usually proximate to the insertion) but can be continuous and, thus, form a compound section at the other extremity (usually the origin; Figs. 3, 4A, 6A).

#### Segmentum mandibularis

The *segmentum mandibularis* of the *adductor mandibulae* inserts on, and is primarily located along, the medial surface of the lower jaw (Fig. 2). Among most teleosts this segment connects at least partially with the *segmentum facialis* via the mandibular tendon. In a few clupeiforms and most neoteleosts parts of the *segmentum mandibularis* may also be associated posteriorly with the buccopharyngeal membrane and its embedded faucal ligament (Fig. 2B). This association is carried further in several eurypterygians in which the faucal ligament serves as the primary site of origin for the *segmentum mandibularis* with the conjunction most pronounced in some synbranchiforms in which the entire segment originates solely from the faucal ligament.

**Figure 9:**
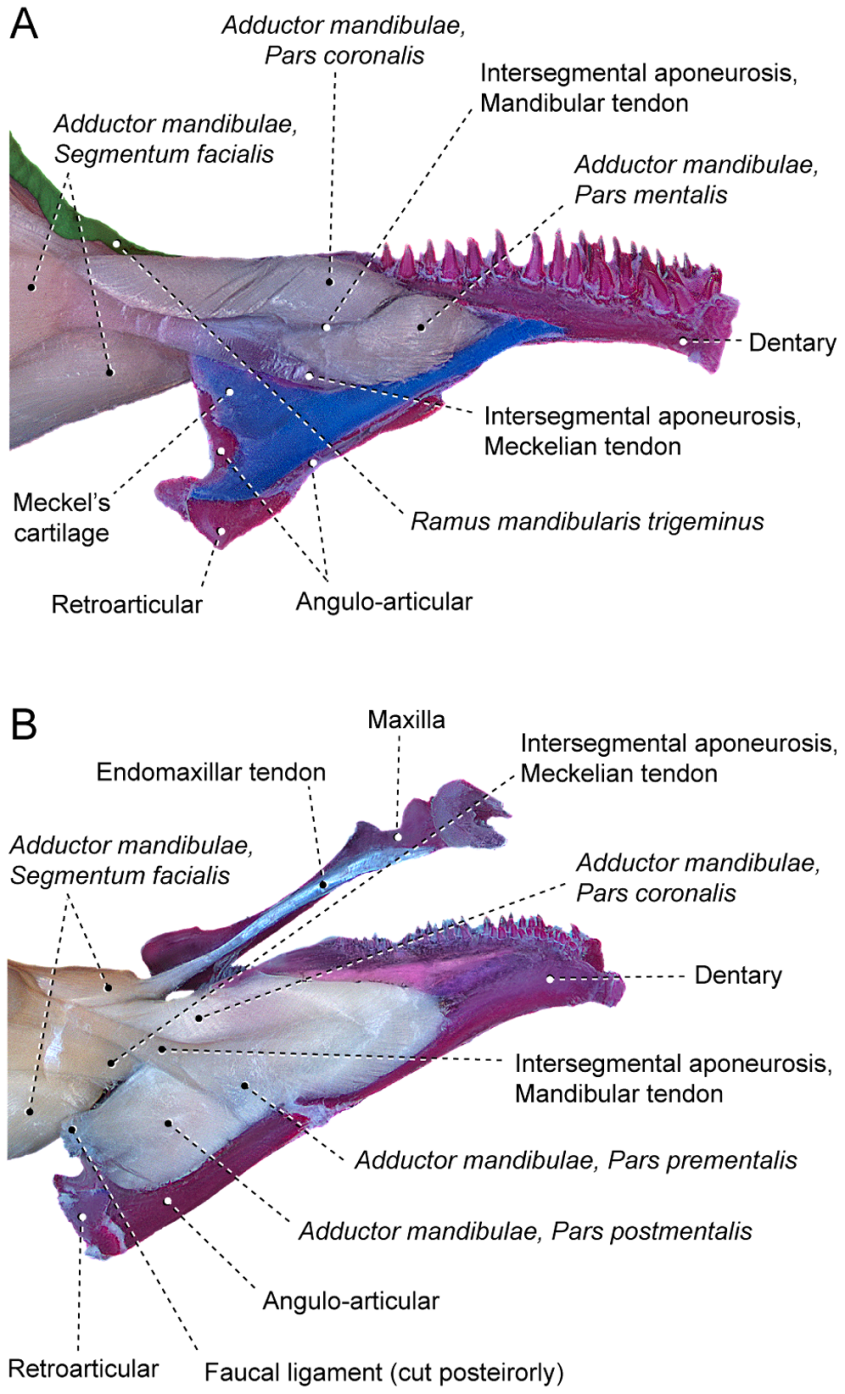
Differentiated mandibular sections. Medial view of left *segmentum mandibularis* and associated structures of (A) *Umbra pygmaea* (Salmoniformes: Umbridae; USNM 333152) and (B) *Anabas testudineus* (Anabantiformes: Anabantidae; USNM 393943). *Ramus mandibularis trigeminus* digitally colored in green.

Some examined groups (*e.g.,* some acanthuriforms, anguilliforms, gonorynchiforms, siluriforms and tetraodontiforms) completely lack the *segmentum mandibularis* (Fig. 8B) whereas that muscle section is present in alternative morphologies in other teleosts. Simplest among these arrangements is that observed in some elopomorphs, osteoglossomorphs and otomorphs in which the *segmentum mandibularis* lacks any trace of differentiation into sections (Fig. 2A). Most teleosts, conversely, have the *segmentum mandibularis* differentiated and subdivided to varying degrees into sections. In most teleosts the *segmentum mandibularis* is nearly bipinnate along its medial face, with the mandibular and/or faucal tendon serving as a central tendinous axis from which most of the muscle fibers arise (Fig. 2B). Since the dorsal and ventral halves of this bipinnate segment are more obviously differentiated and in many instances separated from each other in some taxa (Figs. 3, 9A), it is appropriate to differentiate these muscle portions via distinct names: the dorsal one being termed the *pars coronalis* and the ventral portion identified as the *pars mentalis*. Nevertheless, it is worth mentioning that in most teleosts with a bipinnate *segmentum mandibularis*, these sections are only superficially (medially) distinguishable posteriorly and non-differentiable anteriorly (Fig. 2B).

#### Pars coronalis

This section, which is named in reference to its proximity to the coronoid process of the lower jaw, usually originates from the dorsal part of the mandibular tendon (Figs. 2B, 9). In some elopomorphs, protacanthopterygians, percopsiforms, batrachoidiforms, nototheniiforms and scombriforms the *pars coronalis* is significantly expanded posteriorly beyond the limit of the lower jaw and usually forms a mandibular raphe with the *segmentum facialis* (Fig. 3). The *coronalis* inserts on the portion of the lower jaw dorsal to the Meckel's cartilage; usually on the dentary and angular or any compound bone including the angular (*e.g.*, angulo-articular).

#### Pars mentalis

The *mentalis* whose name is derived from the Latin *mentum*, meaning chin, in reference to its relative position, may extend ventrally beyond Meckel's cartilage and rarely continues caudally beyond the posterior limits of the lower jaw (Fig. 3). This section is more often associated with the faucal ligament than is the *coronalis*. In some aulopiforms, clupeiforms and percomorphaceans the *mentalis* is further differentiated into two subunits, an anterodorsal *prementalis* and a posteroventral *postmentalis* (Fig. 9B). In such configurations, the *prementalis* usually retains an association with the intersegmental aponeurosis and the *coronalis*, whereas the *postmentalis* arises from the faucal ligament and/or the buccopharyngeal membrane.

#### Compound mandibular sections

The *coronalis* and *prementalis* may not be differentiated from one another in some teleosts (*e.g.,* some aulopiforms and stromateiforms). In this configuration these muscle sections form a compound *corono-prementalis*.

### Ramus mandibularis trigeminus

The *ramus mandibularis trigeminus* nerve is a branch of the *truncus infraorbitalis* of the *trigemino-facialis* nerve complex [59], [96]. The path of this muscle has often been considered invariant and, thus, a landmark permitting the identification of facial sections of the *adductor mandibulae* across the Teleostei. Our analysis, in contrast, demonstrates that the course of the nerve towards the inner portion of the lower jaw takes many alternative paths (see Discussion). These include different passages of the *ramus mandibularis trigeminus* lateral, medial or through different sections of the *segmentum facialis* (Table 1).

**Table 1:** Path of *ramus mandibularis trigeminus* nerve relative to facial sections of *adductor mandibulae* muscle among examined specimens.

Path of the *ramus mandibularis trigeminus*		
Internal to	External to	Genera
–	*Segmentum facialis*	*Albula, Chanos^1^, Denticeps, Diaphus, Elops, Maurolicus, Neoscopelus, Oncorhynchus, Osmerus, Pellona*, and *Xenodermichthys*
*Rictalis*	*Malaris* and *stegalis*	*Dactylopterus, Hiodon, Mugil, Raiamas*, and *Xenocharax*
*Ectorictalis* and lateral portion of *endorictalis*	Medial portion of *endorictalis, malaris* and *stegalis*	*Anabas^2^, Atherinella, Carassius^1^, Danio, Rasbora*
*Ectorictalis* and *malaris*	*Endorictalis* and *stegalis*	*Acanthurus, Anabas^2^*
*Rictalis and malaris*	*Stegalis*	*Bathygobius, Brachyhypopomus, Cichla, Elassoma, Fundulus, Gobiesox, Holocentrus, Hypsolebias, Lophius, Lycodes, Megalops, Nototheniops, Osteoglossum, Parexocoetus, Peprilus, Porichthys, Pungitius, Saurida, Synbranchus, Thyrsitops, and Umbra*
*Malaris*	*Rictalis* and *stegalis*	*Caranx, Dules, Hime, Ijimaia, Lutjanus, Orthopristis, Paralichthys, Polymixia, Poromitra, Prionotus, Raneya, Scorpaena, Trachipterus, Triacanthus*, and* Zenopsis*
*Retromalaris*	*Promalaris, rictalis,* and *stegalis*	*Aphredoderus, Merluccius*
*Retromalaris* and *rictalis*	*Promalaris* and *stegalis*	*Percopsis*
*Malaris* and lateral portion of *stegalis*	*Rictalis* and medial portion of *stegalis*	*Antigonia*
*Segmentum facialis*	–	*Scartella*

1 A different and unique nerve path is reported for the same species in the literature (see Discussion);

2 Bilateral asymmetry in the path of the nerve was observed (see Discussion).

## Discussion

### Homologies and evolution

Core to the elucidation of the homologies of the components of the *adductor mandibulae* muscle across the Teleostei is the resolution of two central issues. First among these is the question of how various groups of teleosts come to exhibit different numbers of sections of this muscle. At one extreme of this variation, some groups of teleosts possess only two recognizable muscular components within the *adductor mandibulae,* the *segmenta facialis* and *mandibularis* (some lower teleosts; Fig. 5), whereas other taxa have up to 10 recognizable components of this muscle (*e.g.*, some acanthuriforms and tetraodontiforms; Fig. 8). Two major processes potentially contribute to these discrepancies in component numbers. Under the first of these, the number of sections could be a result of gain and loss of entire sections. The second alternative results in changes in the totality of sections via the subdivision and/or coalescence of sections.

The first of these options, that involving gain and loss of muscle components *in toto*, apparently applies to the whole *segmentum mandibularis*, which analysis revealed to be entirely absent in some acanthuriforms, anguilliforms, cypriniforms, gonorynchiforms, notacanthiforms, osmeroids, osteoglossiforms, siluriforms and tetraodontiforms (pers. obs.; [16]–[18], [20], [22], [23], [25], [62], [64], [97]–[100]). None of these taxa possess muscle fibers that exhibit any of the features characteristic of the *segmentum mandibularis* of other teleosts, *i.e.*, muscle fibers arising from the faucal ligament, intersegmental aponeurosis, or mandibular raphe and inserting on the medial aspects of the lower jaw (Fig. 8).

On the other hand, data from analyzed specimens and literature information support the hypothesis that a process of muscle section division through phylogeny better explains the differences in the number of components within each *segmentum* of the *adductor mandibulae*. Regardless of the presence versus absence of subdivisions within the *segmentum faciali*s, this overall muscle segment typically has a nearly identical area of origin on the suspensorium (compare Figs. 4B, 5B, 6B), a comparable and positionally invariant location relative to adjacent structures (eyeball, buccal membranes, cranial skeleton, other muscles, *etc.*), occupies almost the same portion of the cheek, and invariably inserts on the lower jaw in members of all teleostean orders (Figs. 3, 4A, 5A, 6A). For example, although the *segmentum facialis* is completely undivided in the elopomorph *Elops*, it has: (1) a ventrolateral set of fibers originating from the quadrate and the ventral portion of the preopercle and inserting onto the ventral part of the mandibular tendon; (2) a dorsolateral set of fibers arising from the posteroventral region of the hyomandibula and the dorsal portion of the preopercle and inserting onto the dorsal part of the mandibular tendon and the retrojugal lamina and, thus, indirectly connecting to the maxilla; and (3) a medial set of fibers originating from the metapterygoid and the anterior region of the hyomandibula and inserting on the meckelian tendon (Fig. 5). These very same features are shared, respectively, by the *rictalis*, *malaris* and *stegalis* of the neoteleost *Hime*, although in this taxon these three sets of fibers are anteriorly separated from each other thereby permitting their obvious recognition as distinct muscle subdivisions (Fig. 6).

Given the diversity of jaw architecture across the Teleostei, many members of that clade would be expected to exhibit differences from the basic pattern outlined above. A common alteration involves the attachment of the facial sections to structures in addition to those in the above listing. Such elaborations of these muscles sections are often a function of the expansions of the sections. For example, the *malaris* in most neoteleosts continues further anteriorly over the retrojugal lamina than do the dorsolateral facial muscle fibers which are equivalent to the *malaris* in basal teleosts (compare Figs. 5A with 6A). Some derived neoteleosts carry this particular expansion further (Fig. 7A). Various instances of expansion of portions of the *adductor mandibularis* are followed by subsequent additional subdivisions such as the differentiation of the *malaris* into *promalaris* and *retromalaris* which occurs in several neoteleosts (Fig. 7B). Comparable patterns of muscle expansion and subdivision occur in many other portions of the *adductor mandibulae* across most diverse lineages of teleosts. This renders the examination of a broad comparative sampling of taxa crucial in order to determine the correct homology of each muscle section.

Friel and Wainwright [101] elegantly demonstrated that for tetraodontiforms an evolutionary model of the subdivision of preexisting muscle sections was much more parsimonious than a model based on presumptions of gain and loss of facial sections of the *adductor mandibulae*. The subdivision model to account for differences in the number of facial sections was also implicitly or explicitly adopted in a few other investigations of this muscle in the Teleostei [8], [20], [30], [102]. Further corroborating the subdivision model is ontogenetic data from representatives of diverse groups within the Actinopterygii. Those data demonstrate that the separated facial sections found in the adults of various taxa are ontogenetically derived from the expansion and subsequent sequential subdivision of the single small and undivided muscle mass present in that region earlier in development [31]–[35], [103]. A similar mechanism of serial subdivision recurs repeatedly in many other muscular complexes and underlies the formation of several individual cranial muscles of adult actinopterygians [31], [34], [37], [104]. Notwithstanding this evidence, the vast majority of previous myological studies in the Teleostei involving the *adductor mandibulae*, employ, at least implicitly, a model of the gain and/or loss of sections of that muscle when explaining observed muscles patterns. Terminologies utilized in these discussions amply demonstrate this tendency, with a prime example being the common references to the absence of A_1_ or A_3_ sections (see also discussion under “Alphanumeric Nomenclature – The problems”, below).

**Figure 10:**
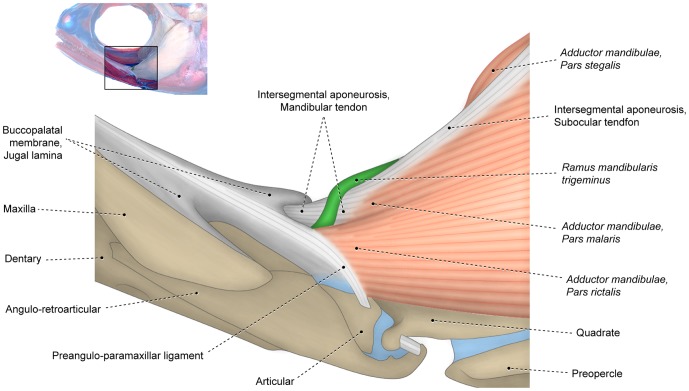
Insertion of *rictalis* on retrojugal lamina. Detail of the region of insertion of *segmentum facialis* of *Hiodon tergisus* (Hiodontiformes: Hiodontidae; USNM 167970) in left lateral view.

Modes of attachment of the *segmentum facialis* to the maxilla are another frequently occurring modification of the *adductor mandibulae* of teleosts. In many lower teleosts (non-Neoteleostei), the entire *segmentum mandibularis* inserts solely on the intersegmental aponeurosis and via that connective tissue sheet onto the lower jaw (Fig. 3). Basal teleosts relatively frequently have a tenuous connection of the *malaris* (or the muscle portion corresponding to this section) with the posterodorsal region of the retrojugal lamina (Fig. 5). As discussed above, an insertion of the *malaris* onto the upper jaw was apparently achieved through this same type of connection in most neoteleosts (Figs. 6, 7). A distinct form of connection of the *segmentum facialis* with the retrojugal lamina is observed in at least one basal teleost, the osteoglossomorph *Hiodon*. In *Hiodon*, the lateral portion of this muscle segment is mostly undifferentiated, except in its anteriormost region where the ventrolateral muscle fibers (presumably corresponding to the *rictalis*) pass lateral to the *ramus mandibularis trigeminus* and insert on the posterolateral portion of the retrojugal lamina, primarily on the preangulo-paramaxillar ligament (Fig. 10). Remaining facial muscle fibers of *Hiodon* converge onto the intersegmental aponeurosis. The *Hiodon* configuration is possibly representative of the mode through which the *rictalis* achieved a connection with the maxilla in many ostariophysans (Fig. 4), osmeroids, smegmamorpharians, anabantiforms and gobiesociforms. In fact, an obvious connection of the *rictalis* with the buccopalatal membrane is evident in many representatives of these groups. *Chanos*, for example, has the tendon that attaches the *rictalis* to the maxilla medially continuous with the buccopalatal membrane (Fig. 4). Several basal characiforms and some osmeroids similarly have at least a partial attachment of the *rictalis* to the lateral portion of the retrojugal lamina, albeit without a direct attachment of that muscle to the maxilla [8].

Part of the *stegalis*, usually the *epistegalis*, may also insert onto the maxilla, as is the case in some acanthuriforms, siluriforms and tetraodontiforms. Among most of the taxa with this form of the insertion, the attachment of a portion of the *stegalis* to the maxilla similarly seems to have arisen via the buccopalatal membrane. Whereas parts of the *stegalis* reach the maxilla directly in some siluriforms, in other members of the order this attachment is indirect and achieved totally or primarily by way of the buccopalatal membrane [20], [105]. Some acanthuriforms and tetraodontiforms achieve a connection of the *epistegalis* to the maxilla via the endomaxillar ligament (Fig. 8B) which is, in turn, derived from the buccopalatal membrane and as such is comparable to the preceding condition.

The evidence of this study indicates that the attachment of the *segmentum facialis* to the maxilla evolved several times across teleosts and that such an attachment involved alternative sections of the muscle in different taxa. An alternative hypothesis is that an insertion on the maxilla is a conserved feature with a correlated supposition that all muscle sections with this attachment are homologous across different groups of teleosts. Resolution of these alternative hypotheses involves the second central issue essential for our discussion: the determination of homologies when conflicting attributes of similarity are present.

Some authors have operated under the assumption of the primacy of certain morphological attributes for the identification of the homologies among sections of the *adductor mandibulae*. A prime example involves the A_1_ section which as originally defined by Vetter [19] was defined by its superficial position and insertion on the maxilla. More recently, the positional component of the definition has ceased to be applied and an attachment to the maxilla has prevailed as the sole defining attribute for the recognition of an A_1_ across the Teleostei. As a result, even medially positioned muscle sections inserting on the maxilla (often termed A_1_β) have been considered to be derived from a superficial A_1_ section [9], [48], [49], [106]. A similar assumption of the primacy of a particular morphological feature for the determination of homologies involves a second anatomical feature, the path of the *ramus mandibularis trigeminus.* Some authors [13], [16], [48], [50]–[53] have proposed that the path of this nerve served as a reliable landmark for identification of facial sections of the *adductor mandibulae* in the Teleostei. This premise is a likely an extrapolation from the classical study of Luther [107], under which muscle divisions in amphibians were named primarily on the basis of their positions relative to the *ramus maxillaris trigeminus* and *ramus mandibularis trigeminus*.

Our analysis of the *adductor mandibulae* morphology across the Teleostei revealed that dependence on a single morphological attribute as the sole or primary indicator of the homologies of any muscle section could lead to arbitrary and unjustifiable homology proposals. Hypotheses of homology of any morphological character, as in this case the sections of the *adductor mandibulae,* should take into account as many attributes as possible. An informative example involves the attachment described above of facial sections to the maxilla: regardless of their insertions, the *rictalis*, *malaris*, and *epistegalis* exhibit nearly identical respective sites of origin, positions, and relationships with most surrounding structures across all examined teleosts (compare Figs. 3, 4, 5, 6). The only significant differences observed across the different taxa are the insertion on the maxilla of (1) the *rictalis* in some ostariophysans (Fig. 4A), smegmamorpharians, anabantiforms, perciforms and gobiesociforms; (2) the *malaris* in some gymnotiforms, alepocephaloids and most neoteleosts (Fig. 6); and (3) the *epistegalis* in some acanthuriforms (Fig. 8B), siluriforms and tetraodontiforms. The *a priori* assumption that the insertion in the maxilla is a conserved feature at a greater level of phylogenetic generality and, thus, a better indicator of homology, would require an assumption of parallel simultaneous migration of the main bodies of the *malaris*, *rictalis*, and *epistegalis* in each of these groups such that a particular section in one taxon would assume the identical position and sites of origin of another section in the other taxon. This complex series of major morphological modifications is highly improbable and far less parsimonious than the alternative proposal of a simple change in the site of insertion of the *malaris*, *rictalis* and *epistegalis* in each of the groups of taxa in question. Recently published ontogenetic data further contradicts the supposition of stable muscle insertion as required by an *a priori* assumption that an insertion of the muscle is constant across the Teleostei. In an insightful investigation of the ontogeny of the *adductor mandibulae* in different representatives of the Tetraodontiformes, Konstantinidis and Harris [35] demonstrated that distinct, non-homologous facial sections independently acquire attachments to the maxilla in different tetraodontiform taxa.

Comparable reasoning applies to the path of the *ramus mandibularis trigeminus*. An assumption of invariance of the course of the nerve through the *adductor mandibulae* across the Teleostei necessitates highly non-parsimonious hypotheses of homology for some muscle complexes. In one of the more extreme situations, the entire *segmentum facialis* in the blenniiform *Scartella*, which lies fully external to the *ramus mandibularis trigeminus*, would be considered non-homologous with any part of the *segmentum facialis* of many other teleosts, including *Albula* (Albuliformes), *Denticeps* (Clupeiformes), *Diaphus* (Myctophiformes), *Elops* (Elopiformes), *Maurolicus* (Stomiatiformes), *Neoscopelus* (Myctophiformes), *Oncorhynchus* (Salmoniformes), *Osmerus* (Salmoniformes), *Pellona* (Clupeiformes) and *Xenodermichthys* (Argentiniformes) in which the entire *segmentum facialis* is situated fully internal to the same nerve (Table 1). Literature surveys furthermore revealed noteworthy variation in the path of this nerve in closely related taxa with virtually identical muscles morphologies. These include nerve path variation between: (1) different species within a single genus (*e.g., Argentina* [108], [109], *Atherina* [21] and *Umbra* [62], [109]); (2) different individuals of a same species; and (3) even in the left versus right sides of the same specimen (*e.g., Ancistrus* cf. *triradiatus* [37]). Among the material we examined, left versus right asymmetry was found in the path of the *ramus mandibularis trigeminus* of the anabantiform *Anabas testudineus* (Table 1). In examined samples of the gonorynchiform *Chanos chanos* and the cypriniform *Carassius auratus*, the observed path of the nerve differs from that reported for these same species in previous studies. Our observations were that the nerve was external to the *segmentum facialis* versus between the *rictalis* and *malaris* (Howes' [16] A_1_ and A_2_) in *Chanos* and in the middle of the *endorictalis* versus between the *endoricto-malaris* and the *stegalis* (Wu and Shen's [52] A2α and A2β) in *Carassius*.

In light of documented variable positioning of the nerve, some prior systematists explicitly rejected the path of the *ramus mandibularis trigeminus* as a reliable landmark for the determination of homologies of the facial section of the *adductor mandibulae* within the Teleostei [5], [9], [34]. Recent studies similarly demonstrate the fallibility of the paradigm of the concept of an invariant path of the *ramus mandibularis trigeminus* in many other groups of vertebrates [110] including within the Lissamphibia [111], the group for which Luther [107] originally proposed the idea of a conserved nerve path. Notwithstanding the ample evidence concerning variability in the path of this nerve, some authors, nonetheless, continued to operate under the premise that the nerve path was an invariant landmark across all teleosts [13], [16], [48], [50]–[53]. To a degree, the reasoning in these studies usually involves the same steps. First, the invariant nerve paths characteristic of some relatively small groups was assumed to support a hypothesis that the course of the nerve was general at higher levels of inclusiveness within the Teleostei. Based on that postulate, variations in the nerve path reported in other studies were presumed to be a function of the misidentification of muscle sections by prior authors rather than actual differences in the course of the nerve. Acting on that assumption, muscle sections were *ex post facto* re-identified primarily or exclusively on the basis of the nerve path, in order to validate the hypothesized invariance in nerve path position and therefore its utility for identification of muscle components. This entire reasoning is clearly problematic because it involves on the one hand an unsupported generalization and on the other circular reasoning.

Admittedly, the insertion of muscles components on the maxilla and the path of the *ramus mandibularis trigeminus* are often conserved in certain groups (Table 1) and may, thus, be useful for the determination of the homologies of subdivisions of the *adductor mandibulae*. These features, however, should be considered as only two among multiple other possible attributes – sites of origin, position, shape, ontogeny, innervation, relationship with adjoining structures, *etc.* – that must be taken into account in global analysis of muscle homology [110]. Conjoined evaluations of these multiple features allows for the evaluation of the most parsimonious hypothesis of homology – *i.e.*, that which minimizes the required changes and conflicts between the different attributes. Although a common practice in comparative morphological studies, this analytic method has often been neglected in myological studies within the Teleostei.

Examined teleosts, as well as virtually all reliable data available in the literature, demonstrate that the known alterations in the *adductor mandibulae* can be explained by the above outlined evolutionary processes. The most frequent type of evolutionary change observed among teleosts is the occurrence of differentiation and separation (subdivision) of muscle sections. Gain or loss of specific facial or mandibular sections was not detected in any teleost, but the entire *segmentum mandibularis* is absent in several lineages (some acanthuriforms, anguilliforms, cypriniforms, gonorynchiforms, notacanthiforms, osmeroids, osteoglossiforms, siluriforms and tetraodontiforms; Fig. 8B). The second most common evolutionary changes are shifts in insertion sites and expansions of muscle sections. Significant alterations in the sites of origin and, especially, in the overall position of the sections are relatively rare and were detected in only a few lineages (*e.g.*, derived groups within the Aulopiformes, Myctophiformes, and Gadiformes; Datovo and Vari, unpublished data).

### Alphanumeric nomenclature – The problems

Nomenclatural schemes that fail to reflect the primary homologies of the components of the *adductor mandibulae* may be a non-issue or prove merely inconvenient for myological and/or phylogenetic investigations centered on smaller subgroups of the Teleostei. Such imprecise terminology conversely poses serious problems when it comes to homology statements in phylogenetic reconstructions of more inclusive groupings. Our analysis amply demonstrated that the coding of phylogenetically informative characters derived from the sections of the *adductor mandibulae* via the present alphanumeric terminology is virtually impossible across the expanse of teleosts. Progressive modification of the terminology first implemented by Vetter [19] by subsequent authors resulted in serial misconceptions as to the evolution of the *adductor mandibulae* across the Teleostei. A notable example is the A_1_ which was traditionally defined by its insertion on the maxilla; a form of attachment which has in retrospect proved to have arisen independently in various lineages within the infraclass. The consequence of this attachment-centered definition was the designation of non-homologous sections of the *adductor mandibulae* as an A_1_ (see discussion above). Due to the resultant confusion the name A_1_ has been applied to at least the following facial muscle sections:

1. the *rictalis* of characiforms [8], [15], [41], [95], [112], [113], gonorynchiforms [16], mugiliforms [12], [14], [47], [114], synbranchiforms [115]–[118] gasterosteiforms [38], [60], [119], atheriniforms [39], [120], [121], beloniforms [120], cyprinodontiforms [12], [21], [39], [63], [122], [123] and anabantiforms [115];
2. the *ectorictalis* of cypriniforms [6], [18], [19], [33], [52], [124];
3. the *endorictalis* of anabantiforms [43], [125];
4. the *malaris* of osteoglossiforms [100], stomiatiforms [48], aulopiforms [14], [49], [126], stephanoberyciforms [48], [49], zeiforms [13], [14], beryciforms [49], [127], acanthuriforms [13], [14], caproiforms [9], [13], [14], cottiforms [9], [11], [14], [29], [47], [128]–[130], gobiiforms [9], [14], [131], labriforms [9], [30], [38], [71], [76], [132]–[144], lophiiforms [47], nototheniiforms [14], [145], some perciforms [7], [9], [12]–[14], [19], [38], [40], [47], [54], [59], [67], [68], [114], [146]–[152], scombriforms [9], [18], [34], [153], scorpaeniforms [11], [13], [14], [26], [27], [29], [38], [114], [154], [155] and trachiniforms [9], [156]–[158];
5. the *promalaris* of carangiforms [159] and gobiiforms [160];
6. the *retromalaris* of carangiforms [9] and gobiiforms [66].

Given the application of the term A_1_ to multiple sections within the *adductor mandibulae* across diverse teleostean groups, it should follow that the term A_2_ was comparably applied inappropriately to the same, or nearly the same, number of non-homologous structures. In actuality, application of the term A_2_ proved to be even more ambiguous than was the case with A_1_ due to an additional complication. The A_3_ section, which in most cases corresponds to the *stegalis* herein, is often poorly differentiated or indistinguishable from the adjoining lateral section of the *adductor mandibulae* which inserts on the lower jaw (*i.e.*, the A_2_ under the alphanumeric terminology). In such morphologies some authors applied composite identifiers such as A_2_A_3_ in an attempt to reflect the compound nature of the sections inserted on the lower jaw [5], [9], [11], [18], [40], [42], [69], [100], [153], [161]. Poor differentiation of the medialmost facial component of the muscle led the vast majority of authors to, however, incorrectly hypothesize that the A_3_ was absent. Thus, the term A_2_ was applied to both simple and compound facial sections (for examples see discussion in Datovo and Castro [8]). In the absence of any muscular attachment to the maxilla, the A_1_ was also considered absent in most studies. As discussed above, the presumption of such absences is incorrect given that abundant information from comparative morphology and ontogeny clearly demonstrates that facial sections are subdivisions of a same primordial muscle mass, the *segmentum facialis*. An outgrowth of these multiple factors was the application of the term A_2_ to an incredible variety of different portions of the *adductor mandibulae.* These include:

1. the entire *segmentum facialis* of elopiforms [5], clupeiforms [47], argentinoids [108], esocoids [62] and salmonoids [47], [108];
2. the *ricto-malaris* of siluriforms [20], [162] and esocoids [18], [19], [108];
3. the *ricto-stegalis* of stomiatiforms [48], zeiforms [13], [14], [47], nototheniiforms [14], perciforms [146], [163], scorpaeniforms [14] and trachiniforms [156]–[158], [164];
4. the *stego-malaris* of gonorynchiforms [16], characiforms [15], mugiliforms [14], [47] and acanthuriforms [14];
5. the *malaris* of characiforms [8], [41], [95], mugiliforms [12], [114], synbranchiforms [115]–[118], gasterosteiforms [60], atheriniforms [39], [120], beloniforms [120] and anabantiforms [115];
6. the *rictalis* of aulopiforms [49], [126], stephanoberyciforms [48], [49], beryciforms [49], [127], blenniiforms [9], [165], caproiforms [9], [13], [14], cottiforms [9], [11], [29], [47], [128]–[130], [166], gobiiforms [9], [131], labriforms [9], [30], [38], [71], [76], [132], [133], [135]–[141], [143], [144], [167], perciforms [7], [9], [19], [38], [40], [47], [54], [59], [68], [114], [147]–[152], scombriforms [9], [34], [153], scorpaeniforms [9], [11], [26], [29], [38] and trachiniforms [9];
7. the *ectorictalis* of anabantiforms [43], [125];
8. the *endorictalis* of cypriniforms [6], [18], [33], [124].

Most often the term A_3_ was applied to the *stegalis* of the nomenclature herein, but with the name incorrectly applied when the *stegalis* is not clearly differentiated from the other facial sections or when some of its subdivisions insert on the maxilla. The term Aω has been almost invariably used to refer to the whole or part of the *segmentum mandibularis*, although this segment was misidentified as a part of the *segmentum facialis* on a few occasions [5], [24]. We do not enumerate herein the ambiguous uses of the terms A_3_, Aω or those commonly applied to subdivisions of the three primary facial sections (A_1_α, A_1_β, A_2_′, A_2_″, *etc.*) in the literature since the above detailed misapplications of the terms A_1_ and A_2_ amply document the magnitude of the problems involved with the present alphanumeric terminology. It is noteworthy that these nomenclatural ambiguities derive not only from different authors who published across the spectrum of groups in the Teleostei, but on occasion involve different taxa within a single analysis (examples of such cases are discussed in Datovo and Castro [8]).

Ambiguities in the application of the alphanumeric terminology most often derive from unavoidable consequences of misconceptions intrinsic to that system of muscle identification rather than reflecting failures of prior authors in the application of that nomenclature. Most notably, these are a function of the problematic definitions of some sections based on what are in actuality variable traits – an insertion on the maxilla and, for some authors, the relative position of the *ramus mandibularis trigeminus* – in conjunction with the common adoption of an evolutionary pathway of gain and loss of muscle sections. Furthermore, most previous investigations of the *adductor mandibulae* were focused on limited subunits of the Teleostei in which the problems posed by the alphanumeric terminology are much less obvious as a consequence of the narrow range of muscular morphological diversity typical within smaller taxonomic groups.

Two recent studies by Wu and Shen [52] and Diogo and Chardon [51] conversely explicitly endeavored to adjust the alphanumeric terminology to produce a nomenclature supposedly reflecting muscle component homologies for the *adductor mandibulae* across all the Teleostei. Neither achieved that goal. The study by Wu and Shen [52] proposed a terminology largely predicated on the postulated stability of the path of the *ramus mandibularis trigeminus* and secondarily of the site of insertion of the muscle sections, together with the extensive application of a model of repeated gain and loss of muscle sections. The many problems associated with the use of these three misconceptions were discussed in detail above. In light of that, the invalidity of Wu and Shen's [52] proposal is not discussed further.

The nomenclature of Diogo and Chardon [51], conversely, requires in-depth commentary. The terminological scheme advanced by those authors was almost completely based on the proposal of Gosline [50], who hypothesized two alternative pathways of differentiation for the *segmentum facialis*; one in ostariophysans and the second in neoteleosts. Gosline [50] proposed that the entire *segmentum facialis* in the lower teleosts inserted solely on the medial face of the lower jaw. Commencing from this base morphology, the anterodorsal portion of the segment in neoteleosts was proposed to differentiate into a separate section and become attached to the maxilla (thus forming an A_1_). Alternatively, in ostariophysans a ventrolateral portion of the same segment would initially acquire an attachment to the posterolateral region of the lower jaw and, in a more derived evolutionary stage, an attachment to the maxilla. Thus, according to Gosline [50], the neoteleostean pathway of differentiation produced muscle divisions non-homologous from those yielded under the ostariophysan subdivision pattern. As a consequence, Gosline [50] retained the traditional alphanumeric terminology for these muscle sections of the *adductor mandibulae* for the Neoteleostei, but introduced the terms “internal division” and “external division” for the main sections resultant from the ostariophysan pathway of subdivision in order to emphasize the incompatibilities between the ostariophysan and neoteleostean arrangements. Under Gosline's [50] scheme the sections herein treated as *rictalis* and *malaris* in Ostariophysi (Fig. 4) consequently would not be comparable with similarly named sections in the Neoteleostei (Fig. 6).

Although we agree with Gosline [50] that an attachment of a portion of the *adductor mandibular* to the maxilla was acquired independently in each group, this does not imply that the dorsolateral and ventrolateral portions of the *segmentum facialis* of these groups are not comparable, *i.e.*, not primarily homologous. On the contrary, as discussed above, these sections retain the same basic sites of origin, position, and relationship with most surrounding structures not only in ostariophysans and neoteleosts but also in almost all teleostean subgroups (including protacanthopterygians; compare Figs. 3, 4, and 6). These common attributes were not considered informative by Gosline [50], who rather admitted that “emphasis has been placed […] on the insertions rather than on the origins of the cheek sections” (p. 658) and proposed that “the course of the *ramus mandibularis* seems to be a better indicator of cheek sections in the *adductor mandibulae* than has generally been acknowledged” (p. 659). The problems associated with these erroneous assumptions were exhaustively detailed above and are not repeated herein. Furthermore, a broader analysis across teleosts demonstrated that the alternative muscle patterns described by Gosline [50] are notably homoplastic across the Teleostei. For example, many gymnotiforms exhibit a muscle configuration nearly identical to the “neoteleostean pattern” [97] whereas most anabantiforms and smegmamorpharians demonstrate the “ostariophysan pattern” [12], [14], [47], [52], [114], [168], and conditions resembling both patterns are found among different taxa of protacanthopterygians [62], [169]–[171], elopomorphs [64], [172] (Fig. 5) and osteoglossomorphs [100] (Fig. 10). Therefore, the hypothesis that the main muscle divisions of neoteleosts and ostariophysans are not primarily homologous is unsupported.

The nomenclatural scheme of Diogo and Chardon [51] was an attempt to adapt the alphanumeric terminology to the until then largely ignored proposal of Gosline [50]. Under the Diogo and Chardon [51] scheme, the entire undivided *segmentum facialis* of the basalmost teleosts should be termed A_2_, whereas the muscle divisions of neoteleosts should retain the traditional alphanumeric terminology. Alternatively, the two main sections yielded by the supposedly unique subdivision pattern in the Ostariophysi were designated by Diogo and Chardon [51] as the A1-OST ( =  Gosline's [50] external division), which was considered unique to that group and non-comparable with any section among neoteleosteans, and the A_2_ ( =  Gosline's [50] internal division) which was treated as homologous to the neoteleostean A_2_. Ostariophysans could further possess an inner A_3_, which was also treated as homologous to the neoteleostean A_3_, and an outer A0, which would be unique to some ostariophysans [51]. In sum, the proposal of Diogo and Chardon [51] based on a mistaken premise – the hypothesis of unique division of the ostariophysan muscle by Gosline [50] – generates an apparent paradox. Although the pattern of subdivision of the *segmentum facialis* in ostariophysans is proposed to be non-comparable with that of neoteleosts, the sections produced via this process of subdivision are, at the same time, comparable (A_2_ and A_3_) and non-comparable (A1-OST and A0) to those generated via the subdivision in neoteleosts. These shortcomings in conjunction with other erroneous factors such as the definition of muscle sections based on variable attributes (insertion on the maxilla and the path of the *ramus mandibularis trigeminus*) and the adoption of an equivocal evolutionary model assuming the gain and loss of muscle sections, resulted in a totally unsatisfactory terminology. Not to belabor the point, but as an example, reference to only two of the nearly 30 studies dealing with the teleostean *adductor mandibulae* authored by Diogo [51], [173], reveals that the term A_2_ was explicitly used to refer to at least five different portions of the *adductor mandibulae* (or more than one-half of all the different uses of the term A_2_ in all the known preceding literature; see above). These are:

1. the entire segmentum mandibularis of Alepocephalus, Clupea, Denticeps, Elops, Hiodon and Salvelinus;
2. the stego-malaris of Chanos, Cromeria, Danio, Hepsetus and Salminus;
3. the ricto-stegalis of Aulopus;
4. the malaris of Brycon and Diplomystes;
5. the rictalis of Perca.

Furthermore, the two primary original contributions of Diogo and Chardon [51] – the creation of the terms A1-OST and A0 – are ambiguously applied across subsequent studies by the first author. Considering only the two studies mentioned above [51], [173], the term A1-OST was used in these papers to refer to the *endorictalis* of cypriniforms and the *rictalis* of characiforms and siluriforms. Moreover, in Diogo and Chardon ([51]: p. 204), the A0 was defined as the “lateral *adductor mandibulae* section that attaches to the upper jaw in […] all cypriniforms, some characiforms, most gonorynchiforms and a large number of gymnotiforms”. In Diogo [173], the muscle sections exhibiting these very same features are contradictorily designated as A1-OST-L in the gonorynchiforms *Chanos*, *Cromeria* and *Parakneria* ( =  *ectorictalis*), the characiform *Distichodus* ( =  *ectorictalis*) and the gymnotiform *Sternopygus* ( =  *malaris*), whereas the A0 section is inexplicably considered to be “exclusively found” solely in cypriniforms ( =  *ectorictalis*) ([173]: p. 261). In summary, the proposals of Diogo and Chardon [51] and Wu and Shen [52] not only failed in the stated purpose of resolving problems with the preceding alphanumeric terminology, but rather substantially increased the nomenclatural confusion associated with the subunits of the *adductor mandibulae*.

### A new terminology

The sum of the above discussed problems perpetuated across more than a century resulted in a progressively complex alphanumeric terminology for the sections of the *adductor mandibulae* which failed to reflect homologous components – the core critical aim of any naming convention. Symptomatic of the irreparable state of this nomenclatural system was the fact that the *rictalis* in the order Siluriformes has received at minimum 11 different designations despite having the same basic position, origin, and insertion in almost all members of the order. Curiously these identifiers span all the three available terms of the alphanumeric terminology for the facial sections:

1. A_1_ or “lateral fibers of muscle b” in loricariids [25];
2. A1-OST in auchenipterids, callichthyids and diplomystids [51], [174]–[176];
3. A1-OST+A2A3′β in trichomycterids [177];
4. A2′ in trichomycterids [20];
5. A2α in bagrids [52];
6. A_2_A_3_′β in clariids [36], [178]–[180];
7. A2ventral in loricariids [162];
8. Ad_1_ in bagrids [17];
9. *adductor mandibulae superficialis* in sisorids [181];
10. external division in diplomystids [50];
11. *partie latérale* or “muscle a” in silurids [182].

Authors were frequently forced to coin inordinately complex terms (*e.g.*, A1βb″mα [101]) in attempts to apply this unsuitable nomenclature to the many modifications that have occurred across the evolution of the *adductor mandibulae* among teleosts. Since most of the problems associated with the alphanumeric terminology are inherent to mistaken underlying original premises, an adaptation of this nomenclature to reflect the homologies of the *adductor mandibulae* is impossible. Retention of the terms A_1_, A_2_, and A_3_ would only increase nomenclatural confusion, more so post the publications of Diogo and Chardon [51] and Wu and Shen [52].

Confronted with the quandary resultant from the inherent problems with the alphanumeric terminology, it is preferable to create a new terminology for the *adductor mandibulae* to reflect the primary homologies of the components of the muscle across the entire Teleostei. The new nomenclature has the additional advantage of using informative anatomical terms (*e.g.*, *rictalis, malaris*) reflective of the basic position of each muscle component, a definite advantage over the uninformative vague alphanumeric codes in the present naming convention. In this, the new nomenclature parallels the naming conventions applied to most other anatomical systems. Short names were selected for primary muscle components to facilitate combinations into relatively brief composite terms designating compound sections (*e.g.*, *ricto-malaris*) and to allow easy aggregation of prefixes and adjectives to indicate subdivisions (*e.g.*, *ectorictalis, promalaris externa*).We found this nomenclature could be successfully employed without complications in all examined teleosts ranging from the simple architecture of the *adductor mandibulae* in some basal teleosts lacking any trace of differentiation in the *segmentum facialis* (Fig. 5A) to the highly intricate muscles with up to ten subdivisions and the highest numbers of distinct attachment sites as in some derived acanthuriforms (Fig. 8) and tetraodontiforms. Detailed accounts of the modifications in the *adductor mandibulae* muscle among the 53 examined teleostean orders will be provided in future publications. That information will be supplemented with synonymies of the nomenclature applied to sections of the *adductor mandibulae* in the major previous publications involving the Teleostei, as well as analyses of the phylogenetic significance of such modifications (Datovo and Vari, unpublished data).

## Supporting Information

Table S1 Material examined.
(PDF)
