# Supplementary material for: The Jaw Adductor Muscle Complex in Teleostean Fishes: Evolution, Homologies and Revised Nomenclature (Osteichthyes: Actinopterygii)
Source: PLoS One. 2013 Apr 2;8(4):e60846. doi: 10.1371/journal.pone.0060846 (PMC3614958; doi:10.1371/journal.pone.0060846)
Supplement: Table S1 — Material examined. (PDF) [file pone.0060846.s001.pdf]

**Table S1.** Material examined.

| Order | Family | Species | Catalog # | # Specimens | |
|---|---|---|---|---|---|
| | | | | **Total** | **Myology** |
| Acanthuriformes | Acanthuridae | *Acanthurus chirurgus* | MZUSP 48207 | 251 | 2 |
| Albuliformes | Albulidae | *Albula vulpes* | LIRP 7427 | 1 | 1 |
| Amiiformes | Amiidae | *Amia calva*[1] | USNM 64338 | 4 | 1 |
| Anabantiformes | Anabantidae | *Anabas testudineus* | USNM 393943 | 4 | 1 |
| Anguilliformes | Anguillidae | *Anguilla reinhardti* | USNM 311978 | 25+ | 1 |
| Argentiniformes | Argentinidae | *Argentina striata* | MZUSP 17914 | 6 | 1 |
| | Alepocephalidae | *Xenodermichthys copei* | MZUSP 86570 | 19 | 1 |
| Ateleopodiformes | Ateleopodidae | *Ijimaia antillarum* | USNM 157838 | 4 | 1 |
| Atheriniformes | Atherinopsidae | *Atherinella brasiliensis* | LIRP 7527 | 310 | 2 |
| Aulopiformes | Aulopidae | *Hime japonica* | USNM 384078 | 24 | 1 |
| | Synodontidae | *Saurida caribbaea* | MZUSP 10647 | 38 | 2 |
| Batrachoidiformes | Batrachoididae | *Porichthys porosissimus* | MZUSP 46971 | 9 | 1 |
| | | *Thalassophryne nattereri*[2] | MZUSP 47261 | 5 | 1 |
| Beloniformes | Exocoetidae | *Parexocoetus hillianus* | MZUSP 103868 | 7 | 1 |
| Beryciformes | Holocentridae | *Holocentrus ascensionis* | MZUSP 60324 | 21 | 2 |
| Blenniiformes | Blenniidae | *Scartella cristata* | MZUSP 60573 | 48 | 2 |
| Caproiformes | Caproidae | *Antigonia capros* | MZUSP 71623 | 14 | 1 |
| Carangiformes | Carangidae | *Caranx latus* | MZUSP 64344 | 13 | 2 |
| Characiformes | Bryconidae | *Brycon orbignyanus* | LIRP 6003 | 32 | 3 |
| | Citharinidae | *Citharinus latus* | MZUSP 84480 | 17 | 1 |
| | Distichodontidae | *Xenocharax spilurus* | AMNH 230302 | 79 | 2 |
| | | | MZUSP 50358 | 2 | 1 |
| Clupeiformes | Denticeptidae | *Denticeps clupeoides* | MZUSP 84776 | 391 | 2 |
| | Engraulidae | *Setipinna taty* | USNM 265905 | 7 | 1 |
| | Pristigasteridae | *Pellona harroweri* | MZUSP 11364 | 17 | 2 |
| Cottiformes | Zoarcidae | *Lycodes pacificus* | USNM 392993 | 74 | 1 |
| Cypriniformes | Cyprinidae | *Carassius auratus* | MZUSP 112353 | 2 | 2 |
| | | *Danio rerio* | MZUSP 112354 | 4 | 3 |
| | | *Raiamas senegalensis* | USNM 271201 | 12 | 1 |
| | | *Rasbora cephalotaenia* | USNM 330848 | 146 | 1 |
| Cyprinodontiformes | Fundulidae | *Fundulus heteroclitus* | MZUSP 67017 | 10 | 1 |
| | Rivulidae | *Hypsolebias antenori* | MZUSP 38342 | 29 | 2 |
| Dactylopteriformes | Dactylopteridae | *Dactylopterus volitans* | MZUSP 79889 | 7 | 1 |
| Elassomatiformes | Elassomatidae | *Elassoma zonatum* | USNM 112744 | 17 | 3 |
| Elopiformes | Elopidae | *Elops lacerta* | MZUSP 84787 | 6 | 1 |
| | Megalopidae | *Megalops cyprinoides* | USNM 102685 | 12 | 1 |
| Gadiformes | Merlucciidae | *Merluccius hubbsi* | MZUSP 80772 | 19 | 2 |
| Gasterosteiformes | Gasterosteidae | *Pungitius sinensis* | USNM 336886 | 196 | 1 |
| Gobiesociformes | Gobiesocidae | *Gobiesox strumosus* | MZUSP 112355 | 1 | 1 |
| Gobiiformes | Gobiidae | *Bathygobius soporator* | MZUSP 66368 | 97 | 2 |
| Gonorynchiformes | Chanidae | *Chanos chanos* | USNM 173572 | 5 | 1 |
| | | | USNM 347536 | 57+ | 1 |
| Gymnotiformes | Gymnotidae | *Gymnotus carapo* | MZUSP 69391 | 10 | 1 |
| | | *Gymnotus paraguensis*[2] | LIRP 6010 | 44 | 2 |
| | Hypopomidae | *Brachyhypopomus pinnicaudatus* | LIRP 6055 | 11 | 2 |
| | Sternopygidae | *Eigenmannia virescens*[2] | LIRP 395 | 12 | 2 |

| Order | Family | Species | Catalog # | # Specimens | |
|---|---|---|---|---|---|
| | | | | Total | Myology |
| Hiodontiformes | Hiodontidae | *Hiodon tergisus* | USNM 167970 | 10 | 1 |
| Labriformes | Cichlidae | *Cichla* cf. *piquiti* | LIRP 6317 | 26 | 2 |
| Lampridiformes | Trachipteridae | *Trachipterus jacksonensis*[2] | MZUSP 78178 | 1 | 1 |
| | | | MZUSP 80771 | 1 | 1 |
| Lepisosteiformes | Lepisosteidae | *Lepisosteus platostomus*[1] | USNM 54983 | 8 | 1 |
| Lophiiformes | Lophiidae | *Lophius gastrophysus* | MZUSP 85811 | 1 | 1 |
| Mugiliformes | Mugilidae | *Mugil curema* | LIRP 7525 | 76 | 2 |
| Myctophiformes | Neoscopelidae | *Neoscopelus macrolepidotus* | USNM 301041 | 6 | 1 |
| | Myctophidae | *Diaphus dumerilii* | MZUSP 90106 | 42 | 2 |
| Notacanthiformes | Halosauridae | *Halosaurus pectoralis* | USNM 317567 | 7 | 1 |
| Nototheniiformes | Nototheniidae | *Nototheniops larseni* | USNM 301728 | 45 | 1 |
| Ophidiiformes | Ophidiidae | *Raneya fluminensis* | MZUSP 61371 | 5 | 1 |
| Osteoglossiformes | Osteoglossidae | *Osteoglossum ferreirai* | USNM 300966 | 20+ | 1 |
| Perciformes | Lutjanidae | *Lutjanus analis* | LIRP 1866 | 5 | 1 |
| | Haemulidae | *Orthopristis ruber* | LIRP 1396 | 21 | 2 |
| | Centropomidae | *Centropomus parallelus* | MZUSP 104606 | 6 | 1 |
| Percopsiformes | Aphredoderidae | *Aphredoderus sayanus* | MZUSP 55046 | 48 | 1 |
| | Percopsidae | *Percopsis omiscomaycus* | USNM 334972 | 10 | 1 |
| Pleuronectiformes | Paralichthydae | *Paralichthys isosceles* | MZUSP 91684 | 11 | 2 |
| Polymixiiformes | Polymixiidae | *Polymixia lowei* | MZUSP 10053 | 6 | 1 |
| Polypteriformes | Polypteridae | *Polypterus senegalus*[1] | USNM 229760 | 9 | 1 |
| | | *Polypterus* sp.[1] | LIRP 7426 | 2 | 1 |
| Salmoniformes | Esocidae | *Esox americanus* | USNM 237253 | 12 | 1 |
| | Galaxiidae | *Galaxias maculatus* | MZUSP 16600 | 21 | 1 |
| | Osmeridae | *Osmerus mordax* | USNM 395752 | 7 | 1 |
| | Salmonidae | *Oncorhynchus mykiss* | MZUSP 85378 | 9 | 1 |
| | Umbridae | *Umbra pygmaea* | USNM 333152 | 23 | 1 |
| Scombriformes | Sphyraenidae | *Sphyraena obtusata*[2] | MZUSP 37378 | 11 | 1 |
| | Gempylidae | *Thyrsitops lepidopoides* | MZUSP 68473 | 4 | 1 |
| Scorpaeniformes | Scorpaenidae | *Scorpaena plumieri* | MZUSP 67283 | 10 | 1 |
| | Serranidae | *Dules auriga* | MZUSP 70831 | 13 | 1 |
| | Triglidae | *Prionotus* sp. | MZUSP 71680 | 174 | 2 |
| Siluriformes | Diplomystidae | *Diplomystes camposensis*[2] | LBP 3106 | 5 | 1 |
| | | *Diplomystes mesembrinus*[2] | LBP 449 | 2 | 1 |
| | Heptapteridae | *Heptapterus mustelinus* | MZUSP 35648 | 60 | 1 |
| | Nematogenyidae | *Nematogenys inermis* | LBP 1002 | 2 | 1 |
| Stephanoberyciformes | Melamphaidae | *Poromitra capito* | USNM 250603 | 12 | 1 |
| Stomiatiformes | Sternoptychidae | *Maurolicus stehmanni* | MZUSP 80266 | 175 | 2 |
| Stromateiformes | Stromateidae | *Peprilus triacanthus* | MZUSP 112356 | 1 | 1 |
| Synbranchiformes | Synbranchidae | *Synbranchus marmoratus* | LIRP 1934 | 3 | 1 |
| Tetraodontiformes | Monacanthidae | *Stephanolepis hispidus* | MZUSP 60332 | 63 | 2 |
| | Triacanthidae | *Triacanthus biaculeatus* | USNM 147904 | 10 | 1 |
| Trachiniformes | Pinguipedidae | *Pseudopercis numida* | MZUSP 66908 | 14 | 1 |
| Zeiformes | Zeidae | *Zenopsis conchifer* | MZUSP 60330 | 15 | 2 |

[1] Outgroup taxon;
[2] Only partially dissected.
